# A novel approach to study the morphology and chemistry of pollen in a phylogenetic context, applied to the halophytic taxon *Nitraria* L.(Nitrariaceae)

**DOI:** 10.7717/peerj.5055

**Published:** 2018-07-19

**Authors:** Amber Woutersen, Phillip E. Jardine, Raul Giovanni Bogotá-Angel, Hong-Xiang Zhang, Daniele Silvestro, Alexandre Antonelli, Elena Gogna, Roy H.J. Erkens, William D. Gosling, Guillaume Dupont-Nivet, Carina Hoorn

**Affiliations:** 1 University of Amsterdam, Institute for Biodiversity and Ecosystem Dynamics (IBED), Amsterdam, The Netherlands; 2 University of Potsdam, Institute of Earth and Environmental Science, Potsdam, Germany; 3 University of Münster, Institute of Geology and Palaeontology, Münster, Germany; 4 Universidad Distrital Francisco José de Caldas, Facultad del Medio Ambiente y Recursos Naturales, Bogotá, Colombia; 5 Key Laboratory of Biogeography and Bioresource in Arid Land, Xinjiang Institute of Ecology and Geography, China Academy of Sciences, Urumqi, China; 6 Gothenburg Global Biodiversity Centre, Göteborg, Sweden; 7 University of Gothenburg, Department of Biological and Environmental Sciences, Göteborg, Sweden; 8 Gothenburg Botanical Garden, Göteborg, Sweden; 9 Harvard University, Department of Organismic and Evolutionary Biology, Cambridge, MA, United States of America; 10 Maastricht University, Maastricht Science Programme, Maastricht, The Netherlands; 11 Université de Rennes, Geosciences Rennes UMR-CNRS, Rennes, France

**Keywords:** FTIR, LM, SEM, Paratethys, Tibet, Sporopollenin, Mediterranean, Steppe-desert, Australia, Palynology

## Abstract

*Nitraria* is a halophytic taxon (i.e., adapted to saline environments) that belongs to the plant family Nitrariaceae and is distributed from the Mediterranean, across Asia into the south-eastern tip of Australia. This taxon is thought to have originated in Asia during the Paleogene (66–23 Ma), alongside the proto-Paratethys epicontinental sea. The evolutionary history of *Nitraria* might hold important clues on the links between climatic and biotic evolution but limited taxonomic documentation of this taxon has thus far hindered this line of research. Here we investigate if the pollen morphology and the chemical composition of the pollen wall are informative of the evolutionary history of *Nitraria* and could explain if origination along the proto-Paratethys and dispersal to the Tibetan Plateau was simultaneous or a secondary process. To answer these questions, we applied a novel approach consisting of a combination of Fourier Transform Infrared spectroscopy (FTIR), to determine the chemical composition of the pollen wall, and pollen morphological analyses using Light Microscopy (LM) and Scanning Electron Microscopy (SEM). We analysed our data using ordinations (principal components analysis and non-metric multidimensional scaling), and directly mapped it on the Nitrariaceae phylogeny to produce a phylomorphospace and a phylochemospace. Our LM, SEM and FTIR analyses show clear morphological and chemical differences between the sister groups *Peganum* and *Nitraria*. Differences in the morphological and chemical characteristics of highland species (*Nitraria schoberi, N. sphaerocarpa, N. sibirica* and *N. tangutorum)* and lowland species (*Nitraria billardierei* and *N. retusa)* are very subtle, with phylogenetic history appearing to be a more important control on *Nitraria* pollen than local environmental conditions. Our approach shows a compelling consistency between the chemical and morphological characteristics of the eight studied Nitrariaceae species, and these traits are in agreement with the phylogenetic tree. Taken together, this demonstrates how novel methods for studying fossil pollen can facilitate the evolutionary investigation of living and extinct taxa, and the environments they represent.

## Introduction

**Figure 1:**
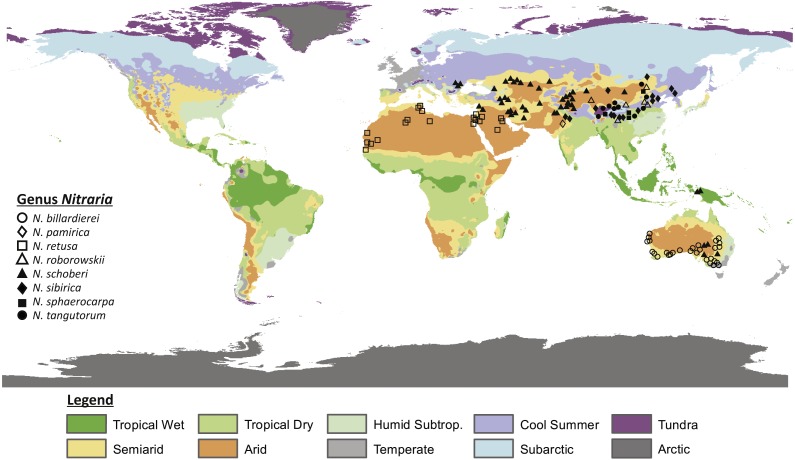
Map showing the present distribution of the genus *Nitraria*. The climate map was modified after Peel, Finlayson & McMahon (2007). Coordinates of the occurrences obtained from GBIF and Tropicos can be found in Appendix S1.

The steppe biome occurs all around the world, covering large areas in Africa, the Middle East, Australia and Eurasia (see ‘Desert and Xeric Shrublands’ and ‘Mediterranean Forests, Woodlands and Scrubs’ distribution in Olson et al., 2001; Fig. 1). In particular, the origins and development of the Eurasian steppe are of great interest since they have been influenced by—and therefore may be a tracer of—past Asian monsoon intensification and inland drying of central Asia since the Paleogene. The Asian steppes are thought to have formed in response to the India-Asia collision, Tibetan Plateau uplift, the retreat of the epicontinental proto-Paratethys sea (which is called the Paratethys sea from 33.9–28.1 Ma; Popov et al., 2004) and global cooling (e.g., Han et al., 2016; Bosboom et al., 2014; Bougeois et al., 2018). Although these processes have shaped present-day population structures and distributions, little is known about the evolution of the Tibetan steppe environment.

Palynological records suggest that the taxonomic composition in the Tibetan region during the Paleogene is distinctly different from Present and in the Paleogene the Tibetan steppe is characterized by a predominance of shrubs such as Ephedraceae and Nitrariaceae (Wang, 1990; Hoorn et al., 2012; Han et al., 2016). How the steppe-desert evolved through time is insufficiently known and a better understanding of the ancestral steppe taxa may therefore help us to clarify the overall evolution of the Tibetan steppe environment.

Nitrariaceae is a family of flowering plants within the order Sapindales that are common in arid climates (Noble & Whalley, 1978; APG, Angiosperm Phylogeny Group; Sheahan, 2010). The family includes the genera *Peganum*, *Tetradiclis* and *Nitraria*. *Peganum* occurs in arid to semiarid parts of Africa, the Middle East and central Asia and has been introduced in the United States, Mexico and Australia (Abbott, Lepak & Daniel, 2007; Zhao et al., 2011). Peganum currently comprises four accepted species (*Peganum harmala, P. nigellastrum, P. mexicanum* and *P. multisectum; The Plant List, 2013*) with *Peganum harmala* being well known because of its medicinal properties (Moloudizargari et al., 2013; Niroumand, Farzaei & Amin, 2015). *Tetradiclis tenella* is the only accepted species within the genus *Tetradiclis* (The Plant List, 2013) and is mainly distributed in the Middle East (GBIF, 2018). *Nitraria* comprises seven accepted species, namely: *Nitraria pamirica*, *N. retusa, N. roborowskii, N. schoberi, N. sibirica, N. sphaerocarpa,* and *N. tangutorum* (The Plant List, 2013). The distributional range of this taxon is Central Asia, southern Europe, and west to north Africa. However, the genus is also present in Papua New Guinea and Australia (Fig. 1) where it is reported as *N. schoberi* and *N. billardierei* with the latter remaining unresolved (i.e., neither ‘Accepted’ or ‘Synonym’; The Plant List, 2013).

All extant species of *Nitraria* are halophytes (i.e., adapted to saline environments) and tend to grow in coastal regions of the Mediterranean, the Middle-East and Southern Australia. In the Mediterranean and Middle-East region *Nitraria retusa* co-occurs with species like *Artemisia herba-alba, Cousinia stenocephala, Phlomis bruguieri* and *Capparis ovata* (https://www.worldwildlife.org/ecoregions/pa0805) and in Southern Australia *Nitraria schoberi* and *N. billardierei* co-occur, among others, with *Arthrocnemum halocnemoides, Atriplex paludosa, Disphyma blackii* and *Suaeda australis* (State Herbarium of South Australia , 1921-2001). A diversion from this coastal pattern is the occurrence of *Nitraria* in the steppe-desert vegetation of the Tibetan Plateau (Noble & Whalley, 1978; Zhang et al., 2015). This steppe is characterized by Poaceae, Asteraceae, Chenopodiaceae (*Haloxylon ammodendron, Salsola collina, Kalidium foliatum* and *Ceratoides lateens*) and minor amounts of Ephedraceae (*Ephedra przewalski*), and Nitrariaceae among others (Zhou, Sun & Chen, 1990 in Cai et al., 2012).

Based on molecular data, *Nitraria* is believed to have originated along the coast of the proto-Paratethys Sea and in the Tibetan region during the early Paleogene, or perhaps even in the Cretaceous (Temirbayeva & Zhang, 2015; Zhang et al., 2015). From here the taxon is thought to have dispersed westward to western central Asia and Africa (Temirbayeva & Zhang, 2015). Subsequent diversification of modern *Nitraria* is thought to have occurred in the Miocene (8.96 Ma), with a dispersal to Australia only taking place during the Pliocene (2.61 Ma) (Zhang et al., 2015). Nevertheless, the palynological record suggests that *Nitraria* peaked in abundance and taxonomic diversity during the late Cretaceous-Paleogene (Wang, 1990; Hoorn et al., 2012; Miao et al., 2016; Han et al., 2016). The latter is in good agreement with the current consensus on the paleogeographic regional evolution with the India-Asia collision occurring around 50–60 Ma and contributing to the retreat of the proto-Paratethys sea until 35 Ma and the growth of the Tibetan Plateau until today (e.g., Molnar, Boos & Battisti, 2010; Favre et al., 2015; Najman et al., 2017; Bosboom et al., 2017).

The Paleogene pollen floras of today’s Tibetan Plateau area were once dominated by Ephedraceae and Nitrariaceae, but at the end of the Eocene (34 Ma) the two families were gradually replaced by Chenopodiaceae, Poaceae and Asteraceae (Song, Wang & Mao, 2008 in Han et al., 2016; Dupont-Nivet, Hoorn & Konert, 2008; Hoorn et al., 2012). This compositional change coincides with the shift from greenhouse to icehouse conditions during the Cenozoic, which in turn resulted in the aridification of central Asia (DeConto & Pollard, 2003; Dupont-Nivet et al., 2007; Abels et al., 2011). Moreover, the retreat of the proto-Paratethys Sea and the impact of the uplift of the NE Tibetan Plateau might have also played a role in the Eocene-Oligocene vegetation shift (Wang et al., 2012; Bosboom et al., 2014).

*Nitraria* has proven to be an important taxon in tracing the evolution of the Tibetan steppe environment (Wang, 1990), and pollen of extant *Nitraria* were previously documented by Agababian & Tumanian (*in Russian, 1972;* original source not found), Xi & Sun (in Chinese, 1987), Xi & Zhang (in Chinese, 1991), Nurbay & Pan (in Chinese, 2003), Perveen & Qaiser (2006) and Hoorn et al. (2012). Yet there are extensive knowledge gaps in the identification and documentation of extant and fossil *Nitraria* pollen, and only recently a phylogeny of the genus was published by Zhang et al. (2015).

Molecular and palynological data together form a powerful toolset to explore the evolutionary history (Chung, Elisens & Skvarla, 2010; Xie & Li, 2012; Kriebel, Khabbazian & Sytsma, 2017) by constructing a phylomorphospace. In this approach, a projection of the phylogenetic tree is plotted into a morphospace which can lead to a better understanding of the history and direction of morphological diversification within taxa (Sidlauskas, 2008; Stone, 2003).

The aim of this study is to assess whether the morphological, chemical and genetic features of extant *Nitraria* pollen permit us to differentiate between species and could provide keys for identification of fossil pollen. Furthermore, we investigated if morphological and chemical evolution are concomitant with molecular evolution. To resolve the relation between the morphology and distribution of the taxon we document here the ecology and distribution of extant *Nitraria* and present a systematic description of their pollen morphology accompanied by LM and SEM photography. In addition, we present data on the organic chemistry of the pollen wall, which was analysed by means of Fourier Transformed Infrared (FTIR) microspectroscopy. We integrated both the morphological and the chemical data sets with the molecular phylogeny of Zhang et al. (2015), resulting in a phylomorphospace and “phylochemospace”. This direct combination of data types has, to our knowledge, never been used before and allows us to integrate taxonomic and palaeoclimatic information inferred from the chemistry of the pollen wall.

## Materials and Methods

### Distribution of modern Nitraria

Occurrence data and altitudinal range of *Nitraria* were collected from GBIF (https://doi.org/10.15468/dl.yvwv2n) and Tropicos (http://www.tropicos.org; 50126115; 34600191; 50126114; 50126119). ‘Observation-only’ and ‘Preserved specimen’ records were used, in order to reduce false occurrences. Data were obtained for the species *Nitraria billardierei*, *N. retusa, N. roborowskii, N. schoberi, N. sibirica, N. sphaerocarpa* and *N. tangutorum.* No data were available for *N. pamirica*. Coordinates of the data points are given in Appendix S1. Additional occurrences of *N. schoberi* were obtained from Zhang et al. (2015) and the occurrence of *N. pamirica* was obtained from Pan, Shen & Chen (1999). Species distributions of *Nitraria pamirica, N. roborowskii, N. sibirica, N. sphaerocarpa* and *N. tangutorum* published in Fang, Wang & Tang (2011) were also taken into account. Occurrence data were supplemented with altitudinal data of the specimens we obtained ourselves for pollen analysis (Appendix S2).

### Processing method

Plant material was collected from various herbaria and collections (see Appendix S2), but for *N. pamirica* no samples could be obtained. Pollen was collected from anthers and standard acetolysis (Erdman, 1986) was applied to remove the cytoplasmic content and to clean the exine; residues were oven-dried while placed in glycerine. Pollen was mounted in glycerine jelly on slides and sealed with paraffin.

### Palynological description of *Nitraria*

Pollen description in general followed the format proposed for the Northwest European Pollen Flora, which is based on the terminology of Punt et al. (2007). Moreover, Hesse et al. (2009) was also taken into account. This was complemented with extra information when necessary. The description included information on polarity, polar symmetry, equatorial and polar shape, number, position and type of apertures, exine thickness, structure and sculpture. All measurements are given in micrometers (µm) average and maximum and minimum ranges are also presented.

### Light microscopy (LM) and Scanning Electron Microscopy (SEM)

For LM analysis, the pollen was described using a Leitz microscope under 1,000× magnification. In total 20 grains per species were measured in polar and equatorial views. Microphotographs were taken with a Normarski Differential Interference Contrast (DIC) microscope (Bercovici, Hadley & Villanueva-Amadoz, 2009). While taking the photos, the varying *z*-axis was recorded and images were later combined through manual z-stacking in Adobe^®^ Photoshop^®^. This stacking technique combines different layers to provide a sense of depth to the images, with a result comparable to 3D photography. The position of the pollen grains that were photographed in LM are recorded with England Finder (EF) and positions indicated in the figure caption.

SEM images of *Nitraria* were obtained at the Servei de Microscopia (Universitat Autònoma de Barcelona, Spain). After standard acetolysis the pollen were deposited in stubs with a carbon adhesive disc, coated with a mixture composed of Au-Pt and the specimens were scanned with a Zeiss EVO and kV ranging from 5 to 15. We also used Field Emission Scanning Electron Microscopy (FESEM) on a Zeiss Merlin, to obtain microphotography of *Nitraria schoberi*.

In addition, SEM is applied to selected specimens of *Nitraria* at the Microscopy Department of the Faculty of Health, Medicine and Life Sciences, Maastricht University (Maastricht, the Netherlands) using a Philips XL30 Scanning Electron Microscope (Philips, Eindhoven, The Netherlands). In the figure caption, we indicated which photographs were made at Maastricht University (UM) and which at Universitat Autònoma de Barcelona (UAB).

### Morphological analysis

**Figure 2:**
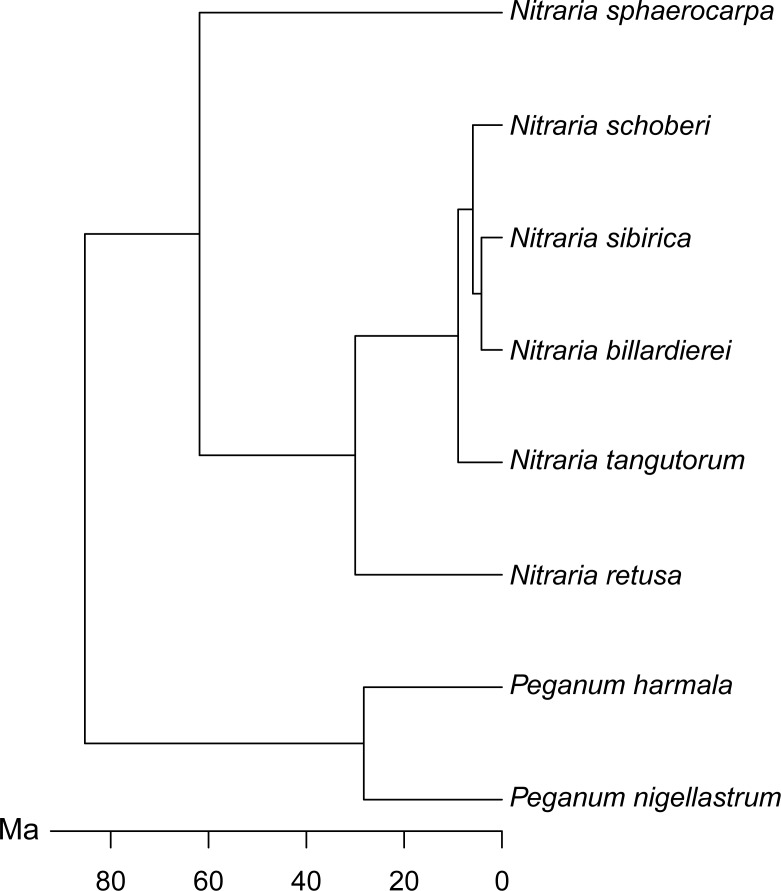
Molecular phylogeny of *Nitraria* and *Peganum,* modified after Zhang et al. (2015).

We compiled a character matrix comprising 24 morphological characters, describing 20 individual specimens from each of the eight *Nitraria* species included in this study ( Supplemental Information 1). We used Gower’s coefficient (Gower, 1971) to calculate dissimilarity between pairs of individuals because the morphological characters are a mix of continuous and discrete variables. We then ordinated the Gower distance matrix using non-metric multidimensional scaling (NMDS), with the number of axes set to two. NMDS seeks to find the optimal arrangement of points in ordination space such that the rank order of dissimilarities is preserved (Kruskal, 1964). To directly relate pollen morphology to the molecular phylogeny of Zhang et al. (2015; Fig. 2) we plotted a phylomorphospace (Sidlauskas, 2008; Hopkins & Smith, 2015). We did this by first calculating the species mean score for each NMDS axis. We then mapped the phylogeny onto the ordination using the ‘phylomorphospace’ function from the R package phytools (Revell, 2012), which plots a projection of the tree into a two-dimensional morphospace (Sidlauskas, 2008; Hopkins & Smith, 2015) using estimated maximum likelihood ancestral character states (here NMDS axis scores) for each internal node on the phylogeny (Revell, 2012; Hopkins & Smith, 2015).

### Chemical palynology

We used FTIR microspectroscopy to characterise the chemistry of the extant *Nitraria* and *Peganum* pollen. FTIR is a vibrational spectroscopic technique that is increasingly being used by palynologists as a taxonomic (Zimmermann, 2010; Steemans et al., 2010; Zimmermann & Kohler, 2014; Bonhomme, Prasad & Gaucherel, 2013; Bağcıoğlu, Zimmermann & Kohler, 2015; Zimmermann et al., 2015; Julier et al., 2016; Zimmermann et al., 2016) and palaeoclimatic (Lomax et al., 2008; Fraser et al., 2014; Lomax & Fraser, 2015; Jardine et al., 2016; Jardine et al., 2017) tool. By directly analysing the chemical signature of palynomorphs it is possible to identify and classify plant taxa with high accuracy (Zimmermann et al., 2016), including distinguishing between closely related species that are morphologically highly similar (Zimmermann, 2010; Julier et al., 2016). FTIR spectroscopy provides a non-destructive and time-efficient way of pollen analysis that can operate at small sample sizes, including down to individual pollen grains (Zimmermann et al., 2015), and can add valuable information to the knowledge obtained by classical morphological analysis (Zimmermann & Kohler, 2014).

Chemical data were generated using a Thermo Scientific Nicolet iN10 MX Dual detector IR microscope at the University of Amsterdam. Individual pollen grains were mounted onto ZnSe windows and analysed using 256 scans and a resolution of 4. We used the same acetolysed pollen residues as for the morphological analysis because acetolysis does not impact upon the pollen wall chemistry (Jardine et al., 2017; Jardine et al., 2015). A background spectrum was taken prior to each measurement and automatically subtracted from the pollen spectrum. The system was purged using a dry nitrogen feed to remove atmospheric CO_2_ and H_2_O variations.

Chemical spectra were generated from individual pollen grains, with a target of 20 grains per species. For both *N. sibirica* and *N. sphaerocarpa* a lack of material meant that this was not possible, and these species are represented by nine and three spectra, respectively. Since no high-quality spectra were obtained for *P. harmala*, this species is not present in the chemical dataset.

The spectra were processed in a number of ways. First, baseline drift was corrected by subtracting a 2nd order polynomial baseline from each spectrum. The spectra were then *z*-score standardised (i.e., so that each had a mean of 0 and a variance of 1) by subtracting the mean spectrum value and dividing by the standard deviation; this removes any differences in absolute absorbance caused by differences in material thickness (Jardine et al., 2015). We also treated the spectra using Savitzky-Golay (SG) smoothing and differentiation (Savitzky & Golay, 1964), since these steps have been shown to improve signal detectability in multivariate chemical analyses (Julier et al., 2016). To find the best combination of spectral processing parameters we treated the spectra in four different ways: no processing, SG smoothing, SG smoothing plus first derivative, and SG smoothing plus second derivative, with the SG smoothing window size varying between 5 and 43. We then used *k-*nearest neighbour (*k-*nn) classification coupled with leave one out cross validation (Varmuza & Filzmoser, 2009; Julier et al., 2016), and calculated the classification success rate (i.e., the percentage of spectra that were correctly classified to species level) for each treatment combination, with the rationale that the parameter combination that produces the best classification success rate will also produce the best separation of taxa in multivariate analyses.

We ordinated the processed spectra using principal components analysis (PCA), which has been widely used with chemometric and environmental data (Varmuza & Filzmoser, 2009). As with other ordination techniques PCA, finds axes of variation so that complex multivariate data can be viewed on a small number of axes. Unlike NMDS, PCA uses absolute values rather than ranked distances, ordinating the data such that the Euclidean distance between objects (in the present case chemical spectra) is preserved (Varmuza & Filzmoser, 2009). The continuous nature of chemical spectral data makes PCA an appropriate ordination technique, and unlike the morphometric data a separate distance matrix does not need to be computed and then ordinated. We also produced a phylochemospace plot by running a PCA on the mean spectrum for each species, and then mapping on the phylogeny following the same procedure as for the phylomorphospace.

All data analyses were carried out using the programme R v.3.4.2 (R Core Team, 2017) with RStudio v.1.0.143 (RStudio Team, 2016), with the packages ape v.5.0 (Paradis, Claude & Strimmer, 2004), baseline v.1.2-1 (Liland & Mevik, 2015), class v.7.3-14 (Venables & Ripley, 2002), FD v.1.0-12 (Laliberté, Legendre & Shipley, 2014) prospectr v.0.1.3 (Stevens & Ramirez-Lopez, 2013), and vegan v.2.4-4 (Oksanen et al., 2017). Datasets and R code are provided in the Supplemental Information 3.

### List of studied species material

*Nitraria billardierei* DC.: New South Wales, South-Western Plains, Australia. *Sources:* Trinity College Dublin-Herbarium (Ireland); Royal Botanic Gardens & Domain Trust and State Herbarium of South Australia, Adelaide, Australia (see Appendix S2). *Slide*: IBED R 8510.

*Nitraria retusa* (Forssk.) Asch.: Northern shore of the Dead Sea, Israel. *Source:* Universitatis Hebraicae Hiersolymitanae (Flora Palaestinae Exciccata) (see Appendix S2); *Slide*: IBED R 8507.

*Nitraria roborowski* Kom.: Slides borrowed from F. Schultz in 2011, and reported in Hoorn et al. (2012). Description from microphotography.

*Nitraria schoberi* L.: South of Teheran, Qom province, Iran. *Source:* Middle East collection, Morteza Djamali (see Appendix S2). *Slide:* Hugo de Vries 6934.

*Nitraria sibirica* Pall.: Toudaohu, Alxa, Inner Mongolia, China. *Source*: Institute of Botany (PE herbarium), Chinese Academy of Sciences, Beijing, China (see Appendix S2). *Slide*: IBED R 8503.

*Nitraria sphaerocarpa* Maxim.: Yinjisha, Kashgar, Xinjiang, China. *Source:* Institute of Botany (PE herbarium), Chinese Academy of Sciences, Beijing, China (see Appendix S2). *Slide:* IBED R 8504.

*Nitraria tangutorum* Bobrov.: Sanshenggong, Alxa, Inner Mongolia, China. *Source:* Institute of Botany (PE herbarium), Chinese Academy of Sciences, Beijing, China (see Appendix S2). *Slide*: IBED R 8505.

*Peganum harmala* var. *multisectum* Maxim.: Guozhigou, Huocheng, Xinjiang, China. *Source*: Institute of Botany (PE herbarium), Chinese Academy of Sciences, Beijing, China (see Appendix S2). *Slide*: IBED R 8506.

*Peganum nigellastrum* Bunge.: Toudaohu, Alxa, Inner Mongolia, China. *Source:* Institute of Botany (PE herbarium), Chinese Academy of Sciences, Beijing, China (see Appendix S2). *Slide:* IBED R 8503.

## Results

### Modern distribution of *Nitraria*

Our data compilation of extant records shows that *Nitraria* is mostly distributed in central Asia, with *N. pamirica, N. roborowskii, N. sphaerocarpa, N. sibirica* and *N. tangutorum* occurring only at the Tibetan Plateau and northwest China (Fig. 1). According to our occurrence data and distributional data from Fang, Wang & Tang (2011), the species *N. roborowskii, N. sphaerocarpa, N. sibirica* and *N. tangutorum* are all well represented across the Tibetan Plateau, whereas *N. pamirica* only occurs in the most northwestern part of China (Fang, Wang & Tang, 2011) and in southwest China (Pan, Shen & Chen, 1999). Furthermore, *N. schoberi* occurs on the Tibetan Plateau as well as in Iran, Papua New Guinea and south Australia. Outside the Tibetan Plateau, *N. billardierei* solely occurs in south Australia and *N. retusa* is restricted to the northern part of Africa. Notably, *N. billardierei* and *N. retusa* occur in coastal (lowland) areas, *N. sibirica* and *N. tangutorum* both occur in lowland and highland environments, and *N. roborowskii, N. schoberi* and *N. sphaerocarpa* are restricted to mountain areas (Table 1).

**Table 1:** Altitudinal range of *Nitraria* and *Peganum*. Range obtained from GBIF and Tropicos and supplemented with altitudes of our obtained specimens.

Species	Altitudinal range (m)
*Nitraria billardierei DC.*	1 – 155
*Nitraria retusa (Forssk.) Asch*	0 – 115
*Nitraria roborowskii Kom.*	910 – 4,402
*Nitraria schoberi L.*	820 – 3,670
*Nitraria sibirica Pall.*	6 – 4,368
*Nitraria sphaerocarpa Maxim.*	912 – 1,867
*Nitraria tangutorum Bobrov.*	497 – 4,560

### Systematic palynology

Below we report on the pollen morphological characterisation of all the taxa surveyed, providing measurements and listing all specimens analysed.

### Family Nitrariaceae Bercht & J. Pres

According to this study and the general pollen characterization of *Tetradiclis* (Sheahan, 2011), pollen of Nitrariaceae is monad, isopolar, and radially symmetric. Pollen shape varies from circular to triangular or quandrangular convex in polar view and elliptic prolate, subprolate to oblate spheroidal or spheroidal in equatorial view. The aperture is tricolporate or hexacolpate (*Tetradiclis*) with a lalongate endoaperture. The exine is tectate or semitectate with a finely striate (*Tetradiclis*), striate, striate perforate or reticulate surface.

### Genus *Nitraria* L.

Pollen is monad, isopolar, radially symmetric. The pollen shape is circular, pseudo-hexagonal, hexagonal or triangular convex in polar view, and elliptic prolate to subprolate in equatorial view. The aperture is tricolporate, and colpi are long and narrow, usually constricted at the equator and have costae colpi and a fastigium. The endoapertures are formed by lalongate pores which are elliptical to rhomboidal in shape. The exine is tectate, nexine thicker than sexine; surface striate to striate-perforate.

**Figure 3:**
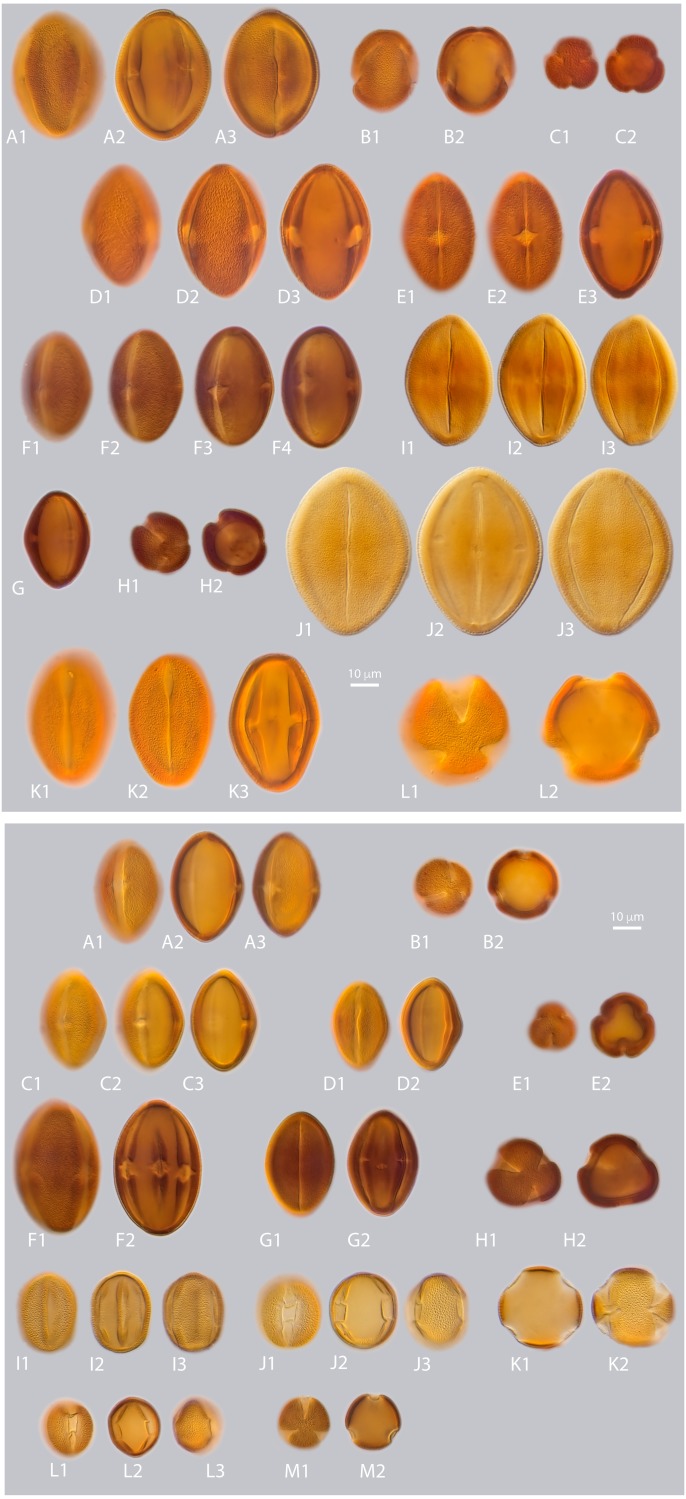
a. LM micrographs of recent *Nitraria* and *Peganum* pollen. Specimen courtesy and England Finder reference are given for each specimen. **(A1-E3) *N. billardierei***, (A1-3) equatorial view (Trinity College Dublin (TCD), Ireland; Q45-1). (B1-2) semipolar view (TCD, Ireland; V38-3). (C1-2) polar view small morphotype (Royal Botanic Gardens & Domain Trust and State Herbarium of South Australia; 43-3). (D1-3) equatorial view at mesocolpus area (Royal Botanic Gardens & Domain Trust and State Herbarium of South Australia; L-M 29-2/4). (E1-3) equatorial view at colporus area (Royal Botanic Gardens & Domain Trust and State Herbarium of South Australia; M29-2). **(F1-H2) *N. retusa*,** (F1-4) equatorial view at mesocolpus and colporus area (Martin Luther Universitat, Germany; N42-2). (G) equatorial view small morphotype (Martin Luther Universitat, Germany; H46-1). (H1-2) polar view (Martin Luther Universitat, Germany; N42). **(I1-J3) *N. roborowskii*,** (J1-3) equatorial view small morphotype, reproduced from Hoorn et al. (2012). (J1-2) equatorial view big morphotype, reproduced from Hoorn et al. (2012). **(K1-L2)** ***N. schoberi*,** (Middle East Pollen Reference Collection (MEPRC), France; K1-3) equatorial view at colporus area (MEPRC, France; EF coordinates not recorded). (L1-2) polar view (MEPRC, France; V19-2). **b. LM micrographs of recent *Nitraria* pollen.** **(A1-B2) *N. sibirica*,** (A1-3) equatorial view (Chinese Academy of Sciences, China; P45-4). (B1-2) polar view (Chinese Academy of Sciences, China; M43-2). **(C1-E2)*N. sphaerocarpa*,**(C1-3) equatorial view at mesocolpus/colporus area (Chinese Academy of Sciences, China; K43-2). (D1-2) equatorial view at mesocolpus area (Chinese Academy of Sciences, China; M44-1). (E1-2) polar view (Chinese Academy of Sciences, China; L42-3). **(F1-H2)*N. tangutorum*,**(F1-2) equatorial view at mesocolpus area (Chinese Academy of Sciences, China; N48-2). (G1-2) equatorial view at colporus area, small morphotype (Chinese Academy of Sciences, China; Q37-2). (H1-2) polar view (Chinese Academy of Sciences, China; H-G 44-1/3). **(I1-K2) *P. harmala*,**(I1-3) equatorial view at colporus-mesocolporus area of a tricolporate grain (Chinese Academy of Sciences, China; L53-1). (J1-3) equatorial view at colporus/mesocolpus area of a tetracolporate grain (Chinese Academy of Sciences, China; E-F 47-2/4). (K1-2) polar view of a tetracolporate grain (Chinese Academy of Sciences, China; G42-2). **(L1-M2) *P. nigellastrum*,** (L1-3) equatorial view at colporus area (Chinese Academy of Sciences, China; Q43-4). (M1-2) polar view (Chinese Academy of Sciences, China; Q43-4).

### Species *Nitraria billardierei* DC.

*LM description. Pollen class:* Tricolporate. Monad, isopolar, radially symmetric, prolate (Fig. 3a A–E). *Aperture*: Tricolporate. Ectoaperture—colpus, long (∼5/6 of polar axis), straight, narrow, equatorially constricted with acute ends; apocolpia asymmetric. Margin (defined as an area of exine around an ectocolpus that is differentiated from the remainder of the sexine, either in ornamentation or by difference in thickness; Punt et al., 2007) observed in polar view, costae colpi and fastigium conspicuous in equatorial view. Endoaperture—porus, lalongate, rhomboidal to elliptic in shape. *Exine*: Tectate; exine slightly thicker in polar areas in relation with the equatorial region; nexine thicker than sexine. Columellae hardly visible, tectum thin. *Sculpture*: Surface striate hardly observed in LM. *Outline*: Polar view circular to pseudo-hexagonal. Equatorial view elliptical. *Measurements*: length: 50.68 (45.8–54) µm; width: 30.38 (28–36.5) µm (see summary in Appendix S3).

**Figure 4:**
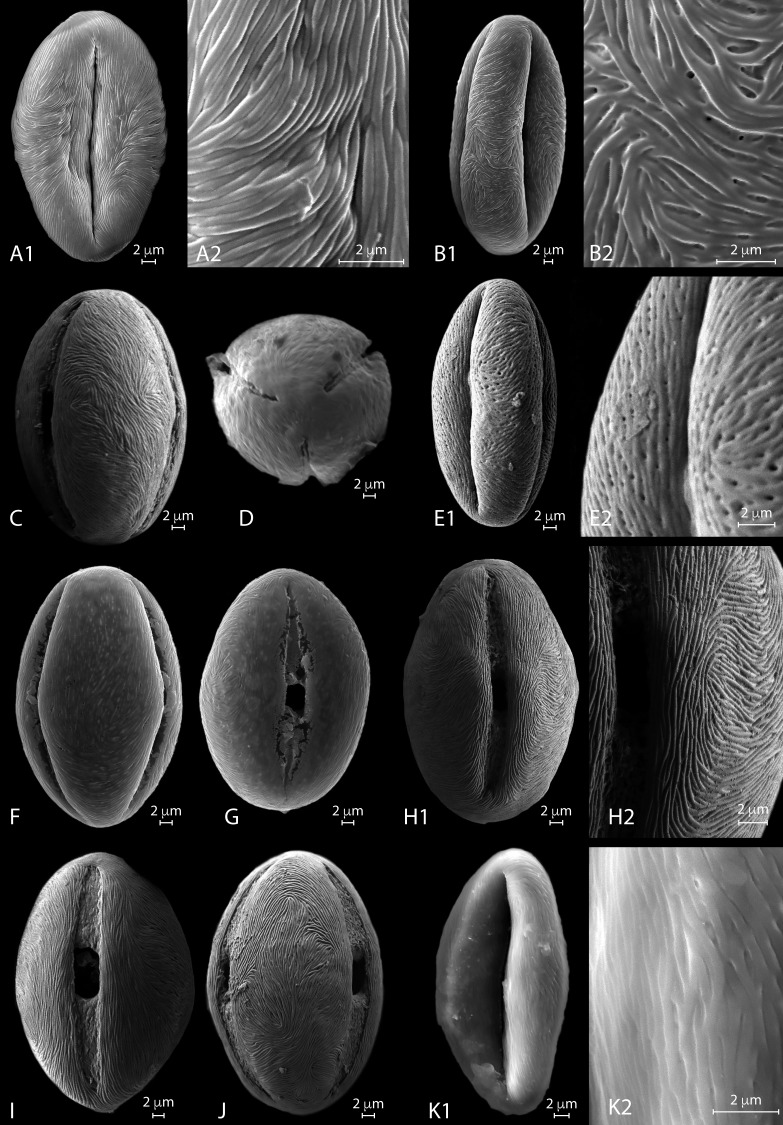
SEM micrographs of extant *Nitraria* pollen. Location of photograph indicated. **(A1-D) *N. billardierei,*** (A1) Equatorial view, note parallel orientation of striae near the colpus (MU). (A2) Detail of “tight” striae (MU). (B1) Equatorial view note transversal striae orientation in mesocolpus area (MU). (B2) note presence of perforations in exine surface (MU). (C) Equatorial view on the mesocolpus area (UAB). (D) Polar view (UAB). **(E1-G) *N*.*retusa*,** (E1) Equatorial view (UAB). (E2) detail of perforation on the exine surface (UAB). (F) Equatorial view on the mesocolpus area (MU). (G) Equatorial view showing colpus and porus (MU). **(H1-J) *N*.*schoberi*,** (H1) Equatorial view clearly showing the colpus, note parallel orientation of the striae near the colpus (UAB; FESEM). (H2) Detail of exine surface, note transversal orientation of the striae in the mesocolpus area (UAB; FESEM). (I) Equatorial view showing colpus and open porus (UAB; FESEM). (J) Equatorial view showing rotation in striae orientation in the mesoculpus area, three main “spirals” can be distinguished (UAB; FESEM). **(K) *N*.*sibirica*,** (K1) Equatorial view (MU). (K2) Detail of the exine surface, note a loose packed striae pattern and some perforations (MU).

*SEM description.* Monads are elliptic, radially symmetric (Fig. 4A). The colpus is almost as long as the polar axis, slightly open; pore eventually conspicuous (Figs. 4B–4C). However, in 4D is it clearly visible that the colpi do not quite meet at the pole. Exine ornamentation is striate with some perforations (Fig. 4B2). Striae are tightly (densely) packed and relatively short. Striae in colpus area are running parallel to the polar axis (Fig. 4A2), whereas in the area of the mesocolpus and apocolpia they are running slightly counter clock direction to slightly perpendicular (Figs. 4A1, 4B1, 4C).

### Species *Nitraria retusa* (Forssk.) Asch.

*LM description. Pollen class:* Tricolporate. Monad, isopolar, radially symmetric, prolate to subprolate (Fig. 3a F–H). *Aperture*: Tricolporate. Ectoaperture—colpus, long (∼7/8 of polar axis), straight, narrow, usually constricted at equator with ends acute; apocolpia asymmetric. Margin observed in polar view, costae colpi and fastigium conspicuous in equatorial view. Endoaperture—porus, lalongate, elliptic to rhomboidal in shape. *Exine*: Tectate; exine slightly thicker in polar areas in relation with the equatorial region; nexine thicker than sexine. Columellae hardly visible, tectum thin. *Sculpture*: Surface striate with some perforations hardly observed in LM. *Outline*: Polar view triangular convex to circular. Equatorial view elliptical. *Measurements:* length: 38.96 (36–42) µm; width: 28.35 (26.8–30) µm (see summary in Appendix S3).

*SEM description.* Monads are elliptic in equatorial view, radially symmetric (Figs. 4E–4G). Colpus almost as long as the polar axis, slightly intruded (margin of colpi pointing inwards) and open; pore conspicuous (Fig. 4G). Exine ornamentation is striate and perforate (Figs. 4E1–4E2). Striae relatively loose packed in the mesocolpia and short, running parallel to the polar axis near the colpus, while running slightly counter clock in the mesocolpus area. Sometimes striae follow perpendicular orientation in the area close to one of the poles in the mesocolpus area (Fig. 4F).

### Species *Nitraria roborowski* Kom.

*LM description. Pollen class:* Tricolporate. Monad, isopolar, radially symmetric, subprolate to prolate (Fig. 3a I-J). *Aperture*: Tricolporate. Ectoaperture—colpus, long (∼5/6 of polar axis), straight, narrow, with ends acute. Costae colpi conspicuous in equatorial view. Endoaperture—porus, lalongate, elliptic shape. *Exine*: Tectate; exine slightly thick. Columellae hardly visible, tectum thin. *Sculpture*: Surface striate with some perforations. *Outline*: Polar view triangular convex. Equatorial view elliptic.

### Species *Nitraria schoberi* L.

*LM description. Pollen class:* Tricolporate. Monad, isopolar, radially symmetric, subprolate to prolate (Fig. 3a K-L). *Aperture*: Tricolporate. Ectoaperture—colpus, long (∼4/5 of polar axis), straight, narrow, commonly constricted at equator with ends acute; apocolpia asymmetric. Margin observed in polar view, costae colpi and fastigium conspicuous in equatorial view. Endoaperture—porus, lalongate, elliptic to rhomboidal in shape. *Exine*: Tectate; exine slightly thicker in polar areas in relation with the equatorial region; nexine thicker than sexine. Columellae hardly visible, tectum thin. *Sculpture*: Surface striate with some perforations hardly observed in LM. *Outline*: Polar view triangular convex to hexagonal. Equatorial view elliptic. *Measurements:* length: 45.49 (42–59) µm; width: 34.71 (30–39) µm (see summary in Appendix S3).

*SEM description.* Monads are elliptic, radially symmetric (Figs. 4H– 4J). Colpus almost as long as the polar axis, open; pore conspicuous (Fig. 4I). Exine ornamentation is striate, no perforations observed (Figs. 4H– 4J). Striae relatively tight and short running parallel to the polar axis near the colpus (Fig. 4I), while running counter clock sometimes forming two to three “spiral” clusters in the equatorial mesocolpus area (Fig. 4J). Striae follow perpendicular orientation in the area close to one of the poles in the mesocolpus area.

### Species *Nitraria sibirica* Pall.

*LM description. Pollen class*: Monad, isopolar, radially symmetric prolate to subprolate (Figs. 3A–3B). *Aperture*: Tricolporate. Ectoaperture—colpus, long (∼7/8 of polar axis), straight, narrow, constricted at equator or not, with ends acute; apocolpia asymmetric. Margin prominent observed in polar view, costae colpi and fastigium conspicuous in equatorial view. Endoaperture—porus, lalongate, ellipsoidal in shape. *Exine*: Tectate; exine slightly thicker in polar areas in relation with the equatorial region; nexine thicker than sexine. Columellae hardly visible, tectum thin. *Sculpture*: Surface finely striate with perforations hardly observed in LM. *Outline*: Polar view pseudohexagonal. Equatorial view elliptic. *Measurements:* length: 39.84 (36–41.8) µm; width: 27.53 (24–29.8) µm (see summary in Appendix S3).

*SEM description*: Monads are elliptic, radially symmetric (Figs. 4K). Colpus almost as long as the polar axis, intruded; pore conspicuous (Fig. 4K1). Exine ornamentation is striate, no perforations observed. Striae relatively loosely packed and short, running fairly parallel to the polar axis near the colpus and in the mesocolpus area (Fig. 4K2).

### Species *Nitraria sphaerocarpa* Maxim.

*LM description. Pollen class*: Tricolporate. Monad, isopolar, radially symmetric prolate to subprolate (Fig. 3b C-E). *Aperture*: Tricolporate. Ectoaperture—colpus, long (∼5/6 of polar axis), straight, narrow, constricted at equator or not, with ends acute; apocolpia asymmetric. Margin observed in polar view, costae colpi and fastigium conspicuous in equatorial view. Endoaperture—porus, lalongate, ellipsoidal in shape. *Exine*: Tectate; exine slightly thicker in polar areas in relation with the equatorial region; nexine thicker than sexine. Columellae hardly visible, tectum thin. *Sculpture*: Surface finely striate with perforations hardly observed in LM. *Outline*: Polar view triangular convex to pseudo-hexagonal. Equatorial view elliptic. *Measurements*: length: 36.66 (34.5–40) µm; width: 25.69 (24–28) µm (see summary in Appendix S3).

**Figure 5:**
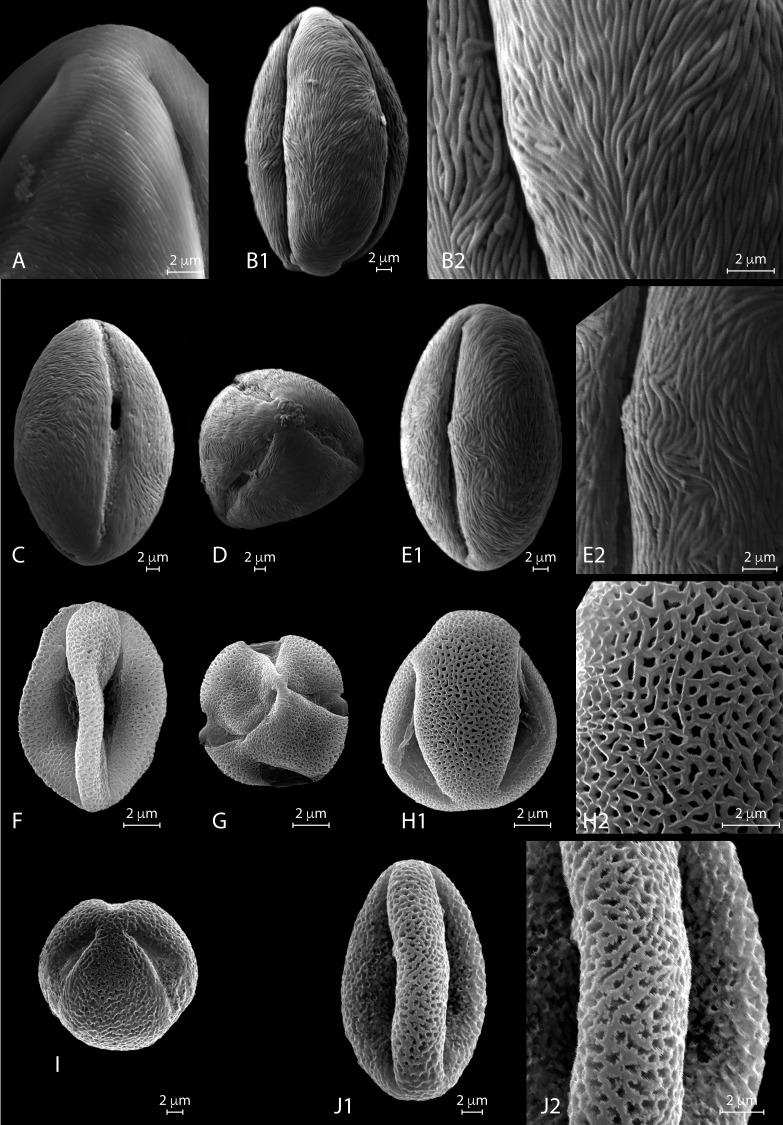
SEM micrographs of extant *Nitraria* and *Peganum* pollen. Location of photograph indicated. **(A-B2) *N*.*sphaerocarpa*,**(A1) Detail of the polar area on an equatorial view (MU). (B1) Equatorial view on mesocolpus area, note parallel orientation of the striae near the colpus and more oblique orientation in the mesoculpus area (UAB). (B2). Detail of the exine surface, striae looks “tight” (UAB). **(C-E2) *N*.*tangutorum*,** (C) Equatorial view showing colpus and open porus, note parallel orientation of the striae near the colpus and transversal orientation on the polar area (UAB). (D) Polar view (UAB). (E1) Equatorial view showing the mesoculpus area (UAB). (E2) detail of the exine surface striae looks “tight” (UAB). **(F-H2) *P*.*harmala*,** (F) Equatorial view (MU). (G) Polar view of a tetracolporate pollen (MU). (H1) Equatorial view (MU). (H2) Detail of the exine surface, note the heterobrochate microreticulate pattern, muri thin in relation to the brochi diameter (MU). **(I-J2) *P*.*nigellastrum*,** (I) Polar view of a tricolporate pollen (MU). (J1) Equatorial view showing a mesoculpus area (MU). (J2) Detail of the exine surface, note the heterobrochate microreticulate pattern, muri thick in relation to the brochi diameter (MU).

*SEM description*. Monads are elliptic, radially symmetric (Figs. 5A–5B). Colpus almost as long as the polar axis, slightly intruded; pore not observed (Fig. 5B1). Exine ornamentation is striate, perforations observed near the colpus. Striae relatively tight and short running parallel to the polar axis near the colpus, while running slightly counter clock in the mesocolpus area (Figs. 5B2, 5B1). Striae follow perpendicular orientation in the area close to one of the poles in the mesocolpus area (Fig. 5A).

### Species *Nitraria tangutorum* Bobrov

*LM description. Pollen class:* Tricolporate. Monad, isopolar, radially symmetric prolate (Fig. 3b F-H). *Aperture*: Tricolporate. Ectoaperture—colpus, long (∼7/8 of polar axis), straight, narrow, occasionally constricted at equator with ends acute; apocolpia asymmetric. Margin observed in polar view, costae colpi and fastigium conspicuous in equatorial view. Endoaperture—porus, lalongate, rhomboidal in shape. *Exine*: Tectate; exine slightly thicker in polar areas in relation with the equatorial region; nexine thicker than sexine. Columellae hardly visible, tectum thin. *Sculpture*: Surface slightly striate with some perforations hardly observed in LM. *Outline*: Polar view triangular convex to circular. Equatorial view elliptic. *Measurements*: length: 42.17 (39–45.5) µm; width: 27.24 (24–29) µm (see summary in Appendix S3).

*SEM description*. Monads are elliptic, radially symmetric (Fig. 5C–5E). Colpus almost as long as the polar axis, slightly intruded and open; pore conspicuous (Fig. 5C). Exine ornamentation is striate, no perforations observed (Fig. 5E1). Striae relatively tight and short running parallel to the polar axis near the colpus (Fig. 5E2), while running slightly counter clock in the mesocolpus area eventually forming a spiral “cluster” in one area close to a pole or running almost parallel. Striae follow perpendicular orientation in the area close to one of the poles in the mesocolpus area (Fig. 5E2).

### Genus *Peganum* L.

Pollen is monad, isopolar, radially symmetric. Pollen shape circular to triangular convex or quadrangular in polar view, and elliptic prolate spheroidal to oblate spheroidal or suboblate in equatorial view. Tricolporate eventually tetracolporate, colpi long open with operculum. Endoaperture—porus lalongate quadrangular in shape. Exine semitectate, sexine slightly thicker than nexine surface microreticulate heterobrochate.

### Species *Peganum harmala* var. *multisectum* Maxim.

*LM description. Pollen class*: Tricolporate. Monad, isopolar, radially symmetric (Fig. 3b I–K). *Aperture*: Tricolporate. Ectoaperture—colpus, long (∼4/5 of polar axis), straight, open, with ends acute; apocolpia asymmetric. Operculum present, margin observed in polar view. Endoaperture—porus, lalongate, quadrangular with outlines not clearly differentiated. *Exine*: Semitectate microreticulate heterobrochate; sexine slightly thicker or not than nexine. Columellae not visible, tectum thin. *Sculpture*: Surface psilate. *Outline*: Polar view circular to triangular convex eventually quadrangular. Equatorial view elliptic oblate spheroidal to suboblate. *Measurements*: length: 24.4 (22–26.5) µm; width: 27.02 (24–29) µm (see summary in Appendix S3).

*SEM description*. Monads are elliptic, radially symmetric (Figs. 5F–5H). Colpus almost as long as the polar axis, intruded, pore not observed (Fig. 5F). Exine ornamentation is microreticulate, heterobrochate (Fig. 5H). Reticulum with lumens decreasing near the colpus and polar areas, and relatively narrow muri (Fig. 5H2).

### Species: *Peganum nigellastrum* Bunge

*LM description. Pollen class*: Tricolporate. Monad, isopolar, radially symmetric (Fig. 3b L-M). *Aperture*: Tricolporate. Ectoaperture—colpus, long (∼4/5 of polar axis), straight, open, with ends acute; apocolpia asymmetric. Operculum present, margin observed in polar view. Endoaperture—porus, lalongate, quadrangular with outlines not clearly differentiated. *Exine*: Semitectate microreticulate heterobrochate; sexine slightly thicker or not than nexine. Columellae not visible, tectum thin. *Sculpture*: Surface psilate. *Outline*: Polar view circular to triangular convex eventually quadrangular. Equatorial view elliptic prolate spheroidal to oblate spheroidal or even spheroidal. *Measurements*:

length: 20.3 (18.5–21.5) µm; width: 20.25 (18.5–21.5) µm (see summary in Appendix S3).

*SEM description*. Monads are elliptic, radially symmetric (Figs. 5I–5J). Colpus almost as long as the polar axis, slightly intruded; pore not observed (Fig. 5J1). Exine ornamentation is microreticulate to perforate (Fig. 5J1), muri heterobrochate. Reticulum with broad muri and lumens irregular in shape and decreasing near the colpus and polar areas (Fig. 5J2).

### Summary of *Nitraria* and *Peganum* pollen morphology

The pollen morphology between *Nitraria* and *Peganum* shows that the tricolporate character of the grains is a shared character. However, *Peganum* differs from *Nitraria* because of its smaller size and its semitectate microreticulate exine. Instead *Nitraria* is tectate to tectate-perforate with a striate exine. A clear distinction between *P*. *harmala* var. *multisectum* and *P*. *nigellastrum* are the irregular borders of the muri in the reticulum of the latter. This character was only observed with SEM.

Measured morphometric characters of *Nitraria* show considerable overlap among the 6 studied taxa (see boxplots: Appendix S4). Our LM observations indicate that the striate pattern varies from faint (*N*. *billardierei*) to striate-perforated or hardly recognizable (*N. schoberi*, *N. retusa, N*. *tangutorum*, *N*. *sphaerocarpa*, and *N*. *sibirica*). Nevertheless, SEM analysis confirms a perforate exine in *N. retusa* and to some degree also in *N.  sphaerocarpa*, in particular near the colpus. A “loose” striate pattern is found in *N. retusa* and to some degree also in *N. sibirica* (seemingly related to a perforate exine) contrary to the “tight” striate pattern in *N. schoberi* and *N. tangutorum.*

**Figure 6:**
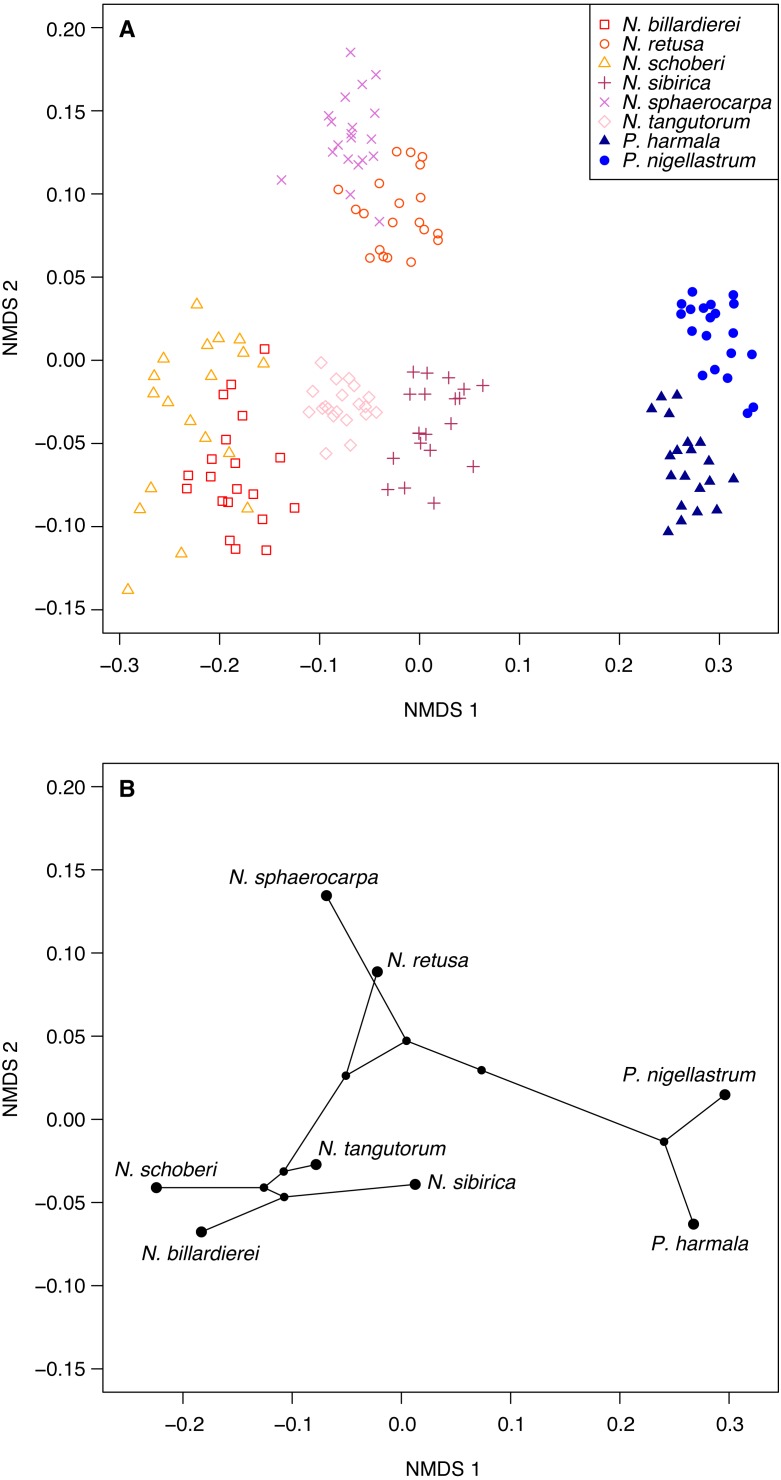
Morphological characteristics of *Nitraria* and *Peganum*. (A) Non-metric multidimensional scaling (NMDS) ordination of *Nitraria* and *Peganum* species morphologies. The ordination is based on pairwise Gower dissimilarities (Gower, 1971) of coded morphological characters for 20 specimens per species. (B) Phylomorphospace based on the mean NMDS axis scores for each species, using the phylogeny of Zhang et al. (2015).

### Multivariate analysis of the pollen morphological data

Ordination of the morphological character data using NMDS shows a clear within-taxon grouping of the specimens, both at the genus and species level (Fig. 6A). NMDS axis 1 primarily separates out the two genera, with some additional separation of *Nitraria* species at the lower end of the axis. Further species-level separation occurs on NMDS axis 2, especially between *N. sphaerocarpa* and *N. retusa* at the upper end of the axis and the other *Nitraria* species at the lower end.

The phylomorphospace (Fig. 6B), where the phylogeny is mapped directly onto the ordination space, demonstrates a phylogenetic signal in the morphological data. This is manifested not only with the separation of *Nitraria* and *Peganum* but also between the early diverging *Nitraria* species *N. sphaerocarpa* and *N. retusa* and those that appeared during the late Miocene to Pliocene *Nitraria* diversification (Zhang et al., 2015).

### Chemical palynology

**Figure 7:**
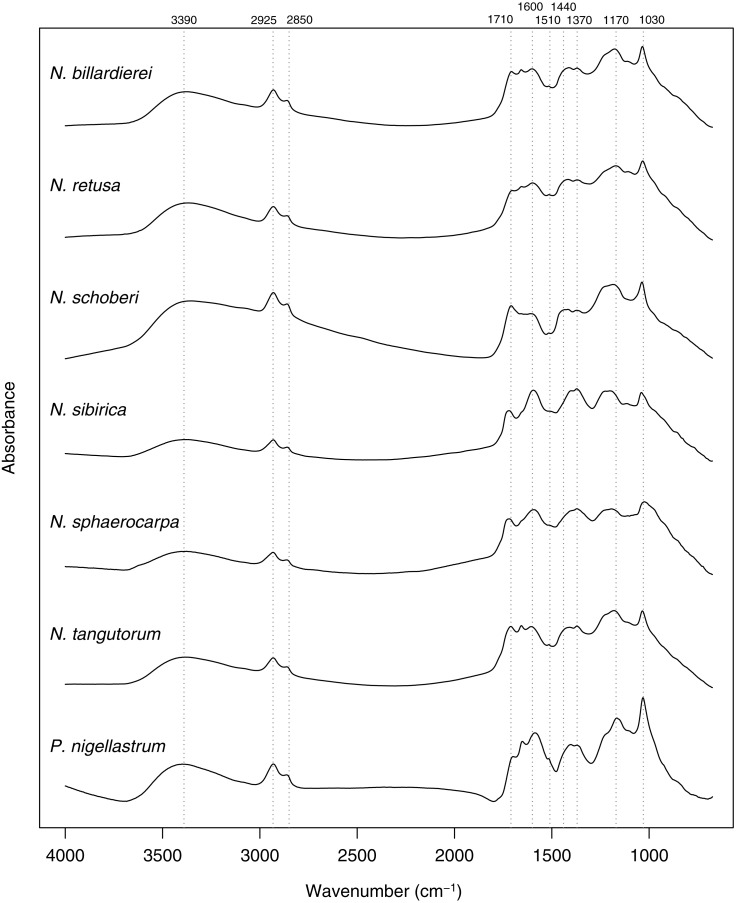
Within-species mean Fourier Transform infrared (FTIR) spectra for *Nitraria* and *Peganum* species. Positions of peaks mentioned in the text are shown as vertical dashed lines with wavenumbers given above.

In common with previous studies of sporopollenin chemistry (e.g., Bağcıoğlu, Zimmermann & Kohler, 2015; Fraser et al., 2012; Jardine et al., 2015; Jardine et al., 2017; Julier et al., 2016), plots of the mean FTIR spectrum for each species show absorbance bands associated with hydroxyl (∼3,400 cm^−1^) and carboxyl (1,710 cm^−1^) groups, aliphatic compounds (2,925 and 2,850 cm^−1^), and aromatic compounds (1,600, 1,510, 1,440, 1,370, 1,170 and 1,030 cm^−1^) (Fig. 7). Principal differences among the spectra relate to the relative heights of these peaks, especially among the carboxyl and aromatic peaks in the region below 1,800 cm^−1^.

The highest classification success rate for the FTIR spectra was 96% (Table 2), which means that ∼108 of the 112 spectra were correctly classified to species level. The combination of processing parameters that produced this classification success rate was SG smoothing plus first or second derivative (see Methods). Both ways of processing the spectra led to highly similar PCA ordinations, and here we present those for the SG smoothing plus second derivative spectra, with the smoothing window size set to 37.

**Figure 8:**
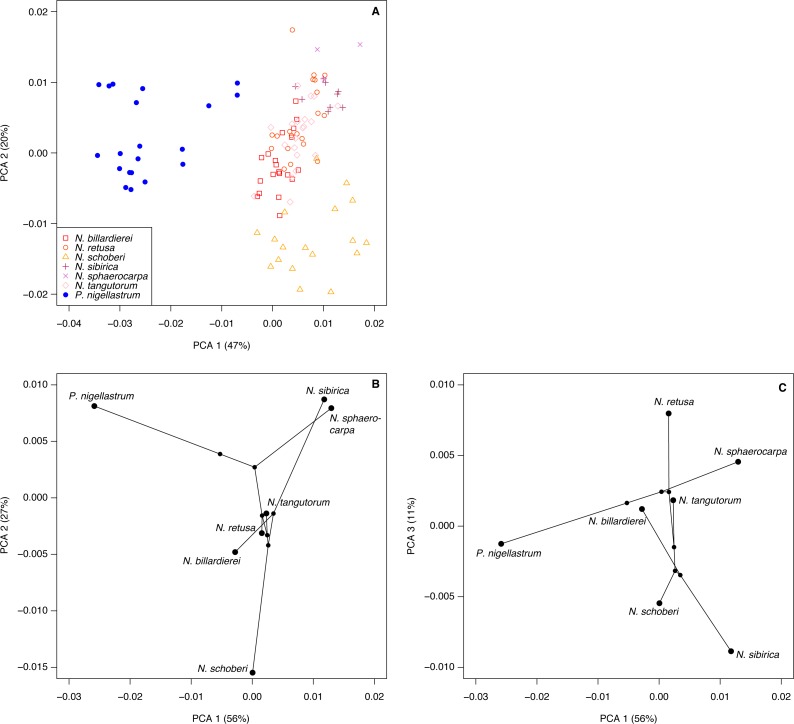
Chemical characteristics of *Nitraria* and *Peganum*. (A) Principal components analysis (PCA) ordination of *Nitraria* and *Peganum* FTIR spectra. (B–C) Phylochemospace based on the mean FTIR spectrum for each species, using the phylogeny of Zhang et al. (2015). Values in parentheses give the percentage of variance in the data explained by each PCA axis.

The first two axes of the PCA of the processed spectra account for 67% of the variation in the dataset (Fig. 8A). *Nitraria* and *Peganum* are separated on the first axis, with the *Nitraria* spectra being distributed across the second axis. The *Nitraria* spectra show within-species groupings, although there is considerable overlap among several of the species in the middle of axis 2. The PCA of the species mean spectra with the phylogeny mapped on shows that while a phylogenetic signal is present in the pollen chemistry it is less clear than with the morphological data, and three PCA axes are needed to reveal the full structure (Figs. 8B–8C). Together these three axes account for 94% of the variation in the chemical data.

## Discussion

### Morphology and chemistry as diagnostic features

The consistency of within-species groupings for both the morphological and chemical data suggest that the multivariate approach used here is useful for assigning fossil specimens to modern taxa, especially following the diversification of extant *Nitraria* species in the late Miocene (Zhang et al., 2015). The 96% classification success rate demonstrated by the chemical data suggests that there is potential to use pollen chemistry, either on its own or in combination with pollen morphology, as a tool for classifying fossil *Nitraria* pollen. However, we acknowledge that this rate was obtained from a relatively small dataset, which means that this is likely an overestimate of true classification success if more individuals representing greater environmental variation were sampled.

*Nitraria* and *Peganum* pollen morphology show a clear phylogenetic structure (Fig. 6). This indicates that fossil specimens can be scored for similar morphological traits to identify extinct lineages and assigned to major lineages within the *Nitraria* phylogeny. This in turn may improve the estimation of divergence times between extant species. In particular, both *N. sphaerocarpa* and *N. retusa* have pollen that has a shorter polar axis than the later diverging taxa, as well as a perforated exine. However, the latter character is only reliably determined with SEM analysis. Even so, this finding suggests that with careful observation, the split between *N. retusa* and the more recently appearing taxa may be discernible from the fossil record. The morphospace inferred for extant taxa also provides the basis for future research to assess morphological disparity (Lupia, 1999) using fossil *Nitraria* specimens.

**Table 2:** Chemical classification success rates from different processing of the spectra.

Treatment	Highest classification success	*w* for highest classification success
Unprocessed	83.9%	–
SG smoothing	84.8%	21 to 43
SG + first derivative	95.5%	9 to 27
SG + second derivative	96.4%	37 and 39

**Notes.**

TITLE SG Savitzky-Golay smoothing w window size for smoothing

Our morphological and chemical data suggest that there are no obvious differences between the modern lowland species (*N. billardierei* and *N. retusa*) and the other *Nitraria* taxa that all occur in highland environments. Similarly, no individual morphological characters appear to distinguish highland and lowland species. Phylogeny appears to be a more important control on *Nitraria* pollen form than the local environmental conditions. A possible environmental or biogeographic signal is recorded on axis 2 of the phylochemospace (Fig. 8B), with the species having positive axis scores (*N. sphaerocarpa*, *N. sibirica*, *P. nigellastrum* and to a lesser extent *N. tangutorum*) represented by specimens from northern China. In particular, *N. sphaerocarpa* and *N. sibirica* have highly similar chemical spectra (Fig. 7). The links between environmental conditions, most obviously UV-B exposure (Jardine et al., 2016), and phylogeny in controlling sporopollenin chemistry require further research, but our results suggest that both play an important role.

### Methodological advances

The chemical analysis aspect of this study is novel for two reasons. First, most studies using extant pollen and spore chemistry for taxonomic or classification purposes have used fresh or herbarium specimens (e.g., Zimmermann, 2010; Zimmermann & Kohler, 2014; Bağcıoğlu, Zimmermann & Kohler, 2015; Zimmermann et al., 2015; Julier et al., 2016; Zimmermann et al., 2016). In these cases, the chemical signal includes proteins, lipids and carbohydrates that are not present in the fossil record where only the sporopollenin is preserved (Jardine et al., 2015; Julier et al., 2016). There is therefore a limited understanding of how closely related taxa will be chemically-distinguishable in fossil samples. Since our study has used isolated sporopollenin following acetolysis, it provides new information on the taxonomic and phylogenetic signal that can be applied to fossil pollen grains.

Second, to our knowledge this is the first time that the phylomorphospace approach has been applied to chemical data of pollen, and the phylogenetic structure present in sporopollenin chemistry directly assessed. Applying a similar approach to larger datasets comprising a greater range of taxa will therefore enable a better understanding of sporopollenin evolution (Fraser et al., 2012), and the phylogenetic underpinnings of the palaeoclimatic and taxonomic indicators that are currently being developed based on pollen and spore chemistry (Zimmermann et al., 2015; Zimmermann et al., 2016).

We found a clear differentiation between species based on pollen morphology and chemical composition, in particular between the earliest diverging species *N. sphaerocarpa* and the more recently derived species *N. schoberi* and *N. billardierei* (Figs. 6 and 8). The two taxa show distinct dispersal patterns: *N. sphaerocarpa* originated in central Asia and is today only found in this region, whereas *N. schoberi* today is also found in Iran, Papua New-Guinea and Australia, and *N. billardierei* is restricted to Australia. The reflection of the biogeographic history of species in the morphological and chemical characteristics of the pollen suggests that either morphological and chemical evolution resulted in, or was driven by, processes related to taxon dispersal. However, further research is required to clarify this relationship.

While the phylogenetic analysis of Zhang et al. (2015) shows that most of the extant *Nitraria* species originated in the late Miocene or Pliocene (ca. 9–5 Ma), the palynological record suggests that diversity was much higher in the Paleocene and Eocene (66–34 Ma) (Wang, 1990; Hoorn et al., 2012; Han et al., 2016). This apparent contradiction calls for future research to focus on understanding this earlier phase in *Nitraria* evolution, including its first appearance and diversification in the proto-Paratethys/Tibetan Plateau region and its subsequent decline in the late Eocene at 34 Ma, and how this fits in with the late Miocene (ca. 9 Ma) diversification and dispersal phase (Zhang et al., 2015). A first step has already been made by Hoorn et al. (2012), who noticed the similarity between fossil *Nitraria* specimens and the earliest diverging *Nitraria* species *N. sphaerocarpa.* The information presented here on the extant species of *Nitraria* provides a valuable comparison dataset for further interrogation of the fossil record and offers the potential to further tie fossil specimens to the extant taxon molecular phylogeny (e.g., Barreda et al., 2015).

## Conclusion

*Nitraria* is an important halophytic taxon of wide current distribution in Asia, Africa and Australia. This taxon has an intriguing past, with predominance in the Paleogene and subsequent dwindling at the end of this period. However, the history of this taxon—and of the Asian steppe biome—is poorly resolved and to address this we looked into novel ways of applying palynology.

In this study we have tested a new method that consists of combining pollen morphological and chemical data sets by producing the phylomorphospace and phylochemospace, in which the palynological data are directly plotted on the Nitrariaceae phylogeny. We conclude that together they form a powerful tool for the identification of modern *Nitraria* species and hold great prospects for exploring the fossil record in general.

Our data indicate that differences between highland and lowland species are subtle and we conclude that phylogenetic history has a more important control on morphology and chemistry of the pollen than local environmental conditions. Future research could focus on the early Paleogene history of this genus and why it went through a major bottleneck at the Eocene-Oligocene Transition. Such study has the potential to provide insights not only on the evolution of the genus, but of the steppe-desert biome as a whole.

##  Supplemental Information

10.7717/peerj.5055/supp-1 Appendix S1 Coordinates of the plotted specimens in Fig. 1, obtained from GBIF, Tropicos or own material described in Appendix S2

10.7717/peerj.5055/supp-2 Appendix S2 Botanical materials. Here we list all the materials that were given on loan from different herbaria to carry out this study

10.7717/peerj.5055/supp-3 Appendix S3 Summary table of the 24 morphological characters used to describe the *Nitraria* and *Peganum* pollen in this study

10.7717/peerj.5055/supp-4 Appendix s4 Boxplots comparing the 18 numerical morphological characters used for pollen morphological analysis between the eight studied species

10.7717/peerj.5055/supp-5 Supplemental Information 1 Measurements of morphological characteristics and FTIR chemical spectra of extant *Nitraria* and *Peganum* pollen (all morphological measurements in µm)

10.7717/peerj.5055/supp-6 Supplemental Information 2 Phylogenetic tree file of Nitrariaceae obtained from Zhang et al. (2015) and used to create the phylomorphospace and phylochemospace

10.7717/peerj.5055/supp-7 Supplemental Information 3 Rcode used for data analysis

## Additional Information and Declarations

### Data Availability

The following information was supplied regarding data availability: The raw data are provided in the Supplemental Files.
