# Supplementary material for: A novel approach to study the morphology and chemistry of pollen in a phylogenetic context, applied to the halophytic taxon Nitraria L.(Nitrariaceae)
Source: PeerJ. 2018 Jul 19;6:e5055. doi: 10.7717/peerj.5055 (PMC6054868; doi:10.7717/peerj.5055)
Supplement: Appendix S2 [file peerj-06-5055-s002.docx]

**APPENDIX 2**

Botanical materials. Here we list all the materials that were given on loan from different herbaria to carry out this study.

1) Institute of Botany (PE herbarium), Chinese Academy of Sciences, Nanxincun 20, Xiangshan, Beijing 100093, China. Courtesy professor Ming-Li Zhang, PE herbarium

- ***Nitraria sibirica* Pall**; Xi-lin Yang 137 (16 June 1962), Toudaohu, Alxa, Inner Mongolia, China
- ***Nitraria sphaerocarpa* Maxim**; PE 00972356; Xinjiang Exp team 00030 (2 may 1959), Yinjisha, Kashgar, Xinjiang, China, alt. 1350 m.
- ***Nitraria tangutorum* Bobrov**; PE 00972381; (4 June 1958), Sanshenggong, Alxa, Inner Mongolia, China, alt. 980 m.
- ***Peganum nigellastrum* Bunge**; Xi-lin Yang 021 (30 May 1963), Toudaohu, Alxa, Inner Mongolia, China
- ***Peganum harmala* var. multisecta Maxim**.; Zhong-tao Wang 327 (25 June 2007), Guozhigou, Huocheng, Xinjiang, China, alt. 1353

2) Martin Luther Universitat, Halle, Saale, Germany. Courtesy professor Uwe Braun.

- ***Nitraria retusa*** *(Forsk.) Aschers.* ex HAL 10213; A. Eig, N. Feinbrun et M.Zohary. Northern shore of the Dead Sea, near Kalia, 4 IV. 1934. Leg. J. Amdursky et J.Olami. (Phytogeogr. value: A sub-West Saharo-SIndian species, penetrating into the Sudano-Deccanian region. One of the most prominent shrubs in the saline places of all three Saaharo-Sindian territories of Palestine (Negueb, the lower Jordan valley), and the southern part of Transjordania, east of its med. Territory). In wadi Sarhan (Transjordania) we recently found a well-developed Nitrarietum retusae, stretching over many kilometres. **Source:** Ver. Bot. Ver. Prov. Brandenb. XVIII (1876), p.94). Labeled: Universitatis Hebraicae Hiersolymitanae (Flora Palaestinae Exciccata)

3) Trinity College Dublin Herbarium, Dublin, Ireland. Courtesy professor Parnell.

- ***Nitraria billardierei****;* TCD0013252, Royal Botanic Gardens & Domain Trust, Sydney, Adelaide, Australia. 3 pollen grains in slide. (Only used for LM photographs).

4) State Herbarium of South Australia, Adelaide, Australia.

- ***Nitraria billardierei* DC.***;* R.G. Coveny AD 119995 (8 October 2000), New South Wales, South-Western Plains Bot. Div.; c. 6 km of Stony Crossing off the Kyalite San Hill Road. Coordinates ex collector: 35^O^ 06’05’’, 143^o^ 31’22’’; precn 2, alt. 86 m (used for SEM photograph).

5) Private collection professor Morteza Djamali, Aix en Provence, France.

- ***Nitraria schoberi****;* H. Akhani 1361, Middle East Pollen Reference Collection (MEPRC) at Aix en Provence; the sample has code MEPRC-441, 34°18'N, 051°19'E, alt. 820 m.

6) F. Schlütz (reproduced from photograph in *Hoorn* *et al. 2012*):

- ***Nitraria roborowskii* Komarov***;* private collection (see *Hoorn et al. 2012* for information)

After extensive effort we had to conclude that we were not able to get materials from the following taxa:

- ***N. pamirica****;* distribution in Kazakhstan, Kyrgyzstan, Tajikistan, Turkmenistan, Uzbekistan (source: <http://www.tropicos.org/Name/34600189>)
- ***N. komarovii****;* distribution in NW-Iran and Iranian Azerbaijan, S-European Russia, C-Asia (source: GBIFID 1096323278)
