# Supplementary material for: A novel approach to study the morphology and chemistry of pollen in a phylogenetic context, applied to the halophytic taxon Nitraria L.(Nitrariaceae)
Source: PeerJ. 2018 Jul 19;6:e5055. doi: 10.7717/peerj.5055 (PMC6054868; doi:10.7717/peerj.5055)
Supplement: Appendix s4 [file peerj-06-5055-s004.pdf]

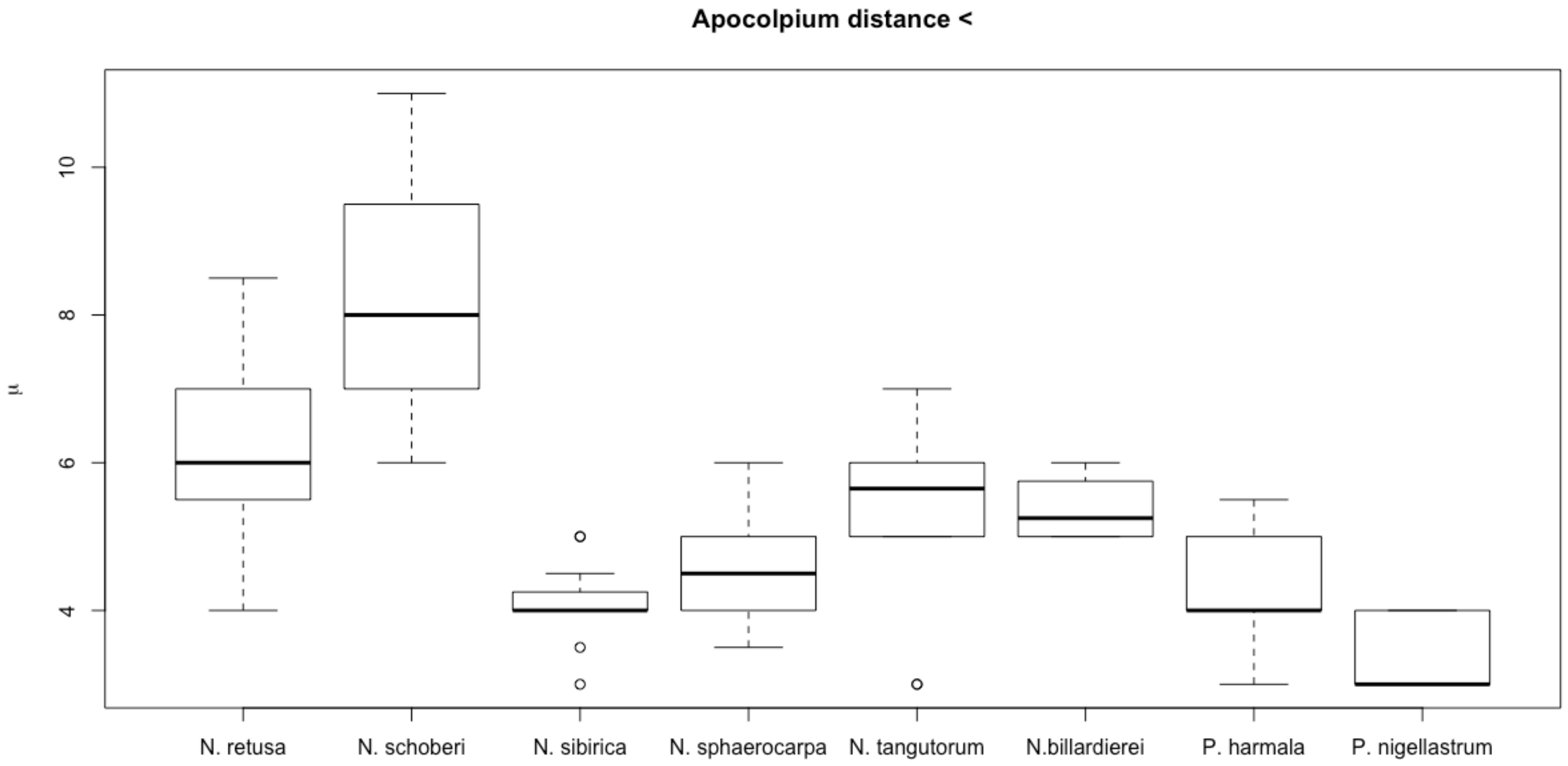
Apocolpium distance <
μ
4
6
8
10
N. retusa
N. schoberi
N. sibirica
N. sphaerocarpa
N. tangutorum
N.billardierei
P. harmala
P. nigellastrum

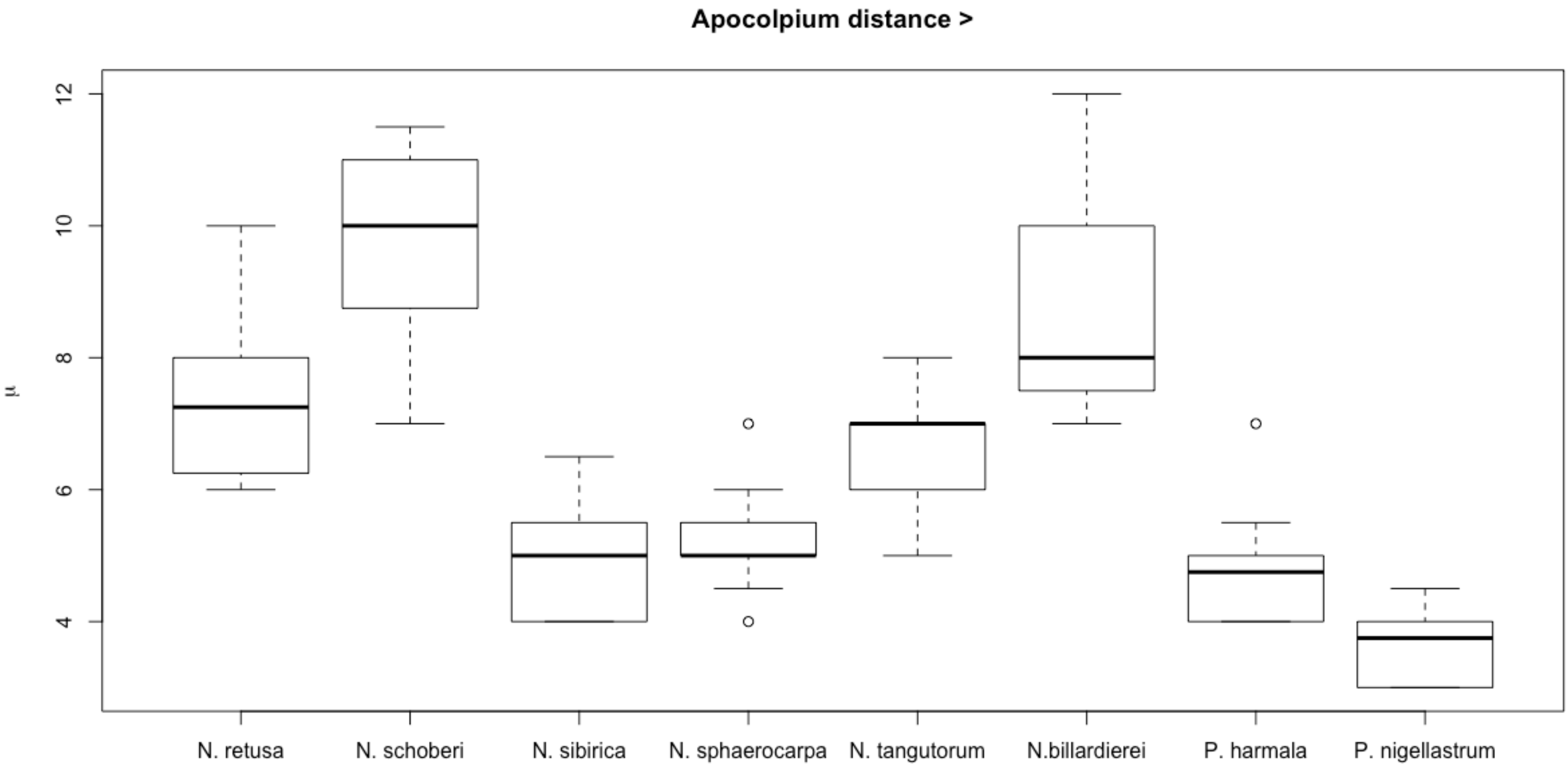
Apocolpium distance >
μ
12
10
8
6
4
N. retusa
N. schoberi
N. sibirica
N. sphaerocarpa
N. tangutorum
N.billardierei
P. harmala
P. nigellastrum

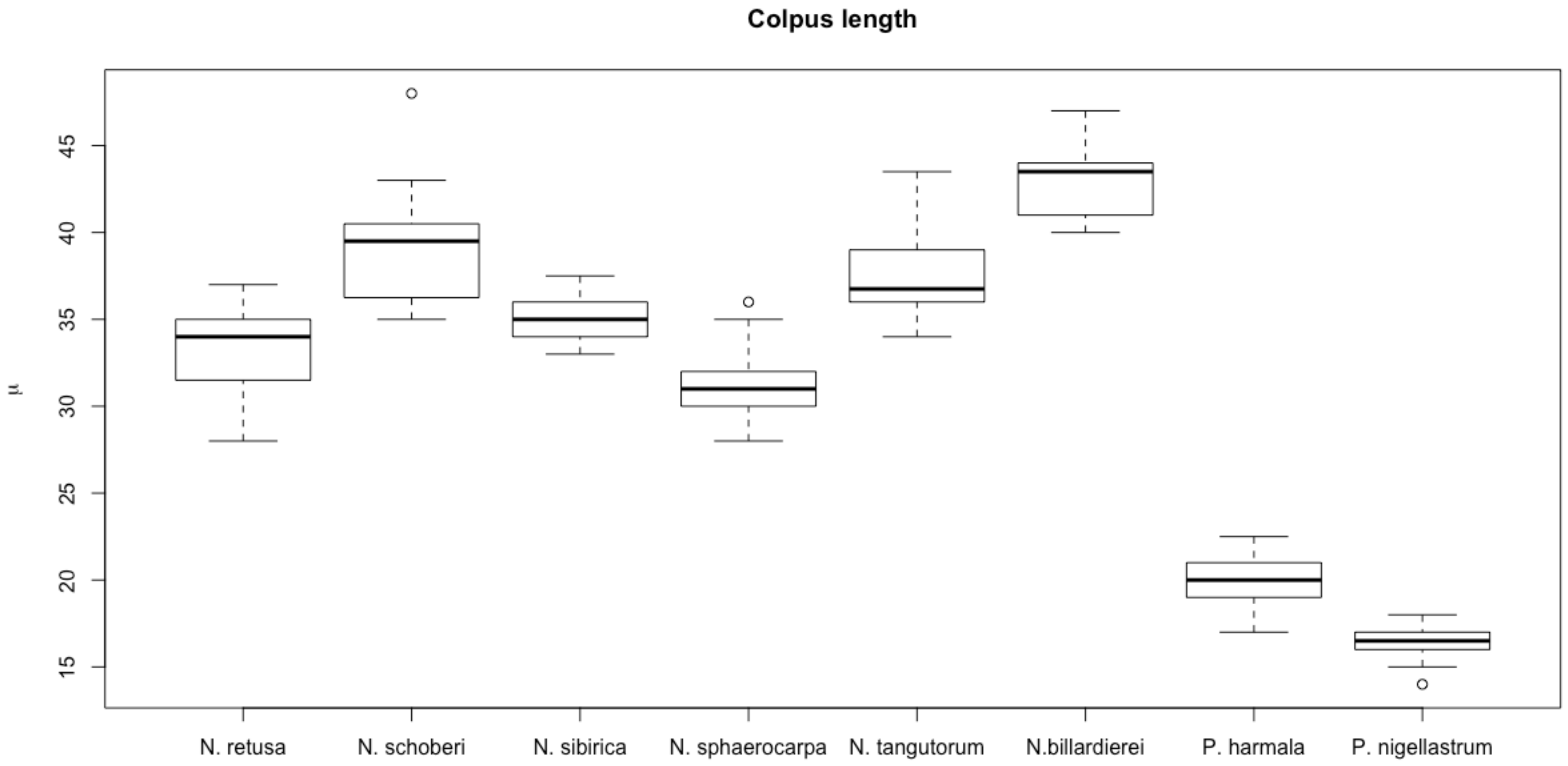
Colpus length
μ
45
40
35
30
25
20
15
N. retusa
N. schoberi
N. sibirica
N. sphaerocarpa
N. tangutorum
N.billardierei
P. harmala
P. nigellastrum

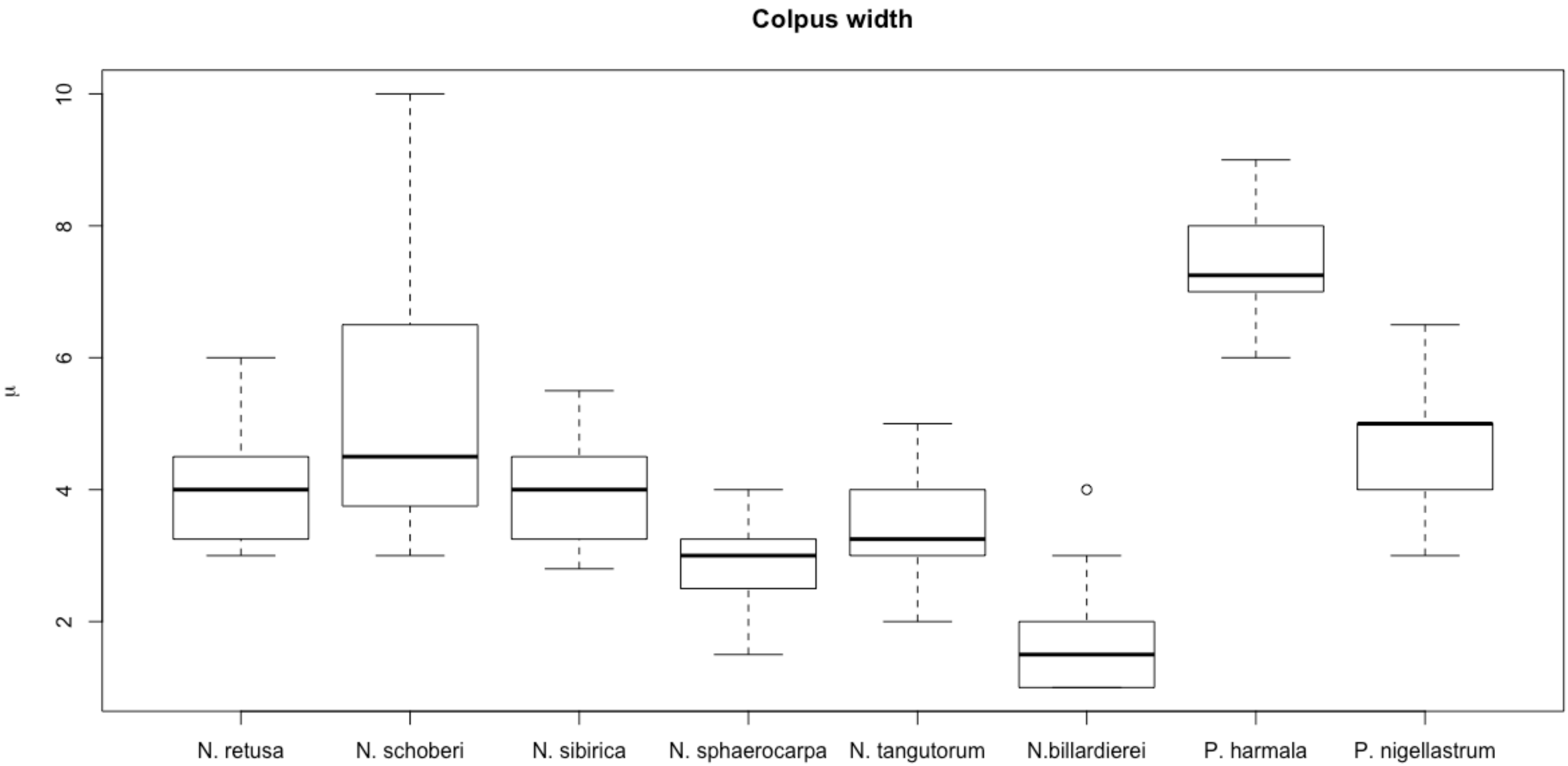

Colpus width
μ
10
8
6
4
2
N. retusa
N. schoberi
N. sibirica
N. sphaerocarpa
N. tangutorum
N.billardierei
P. harmala
P. nigellastrum

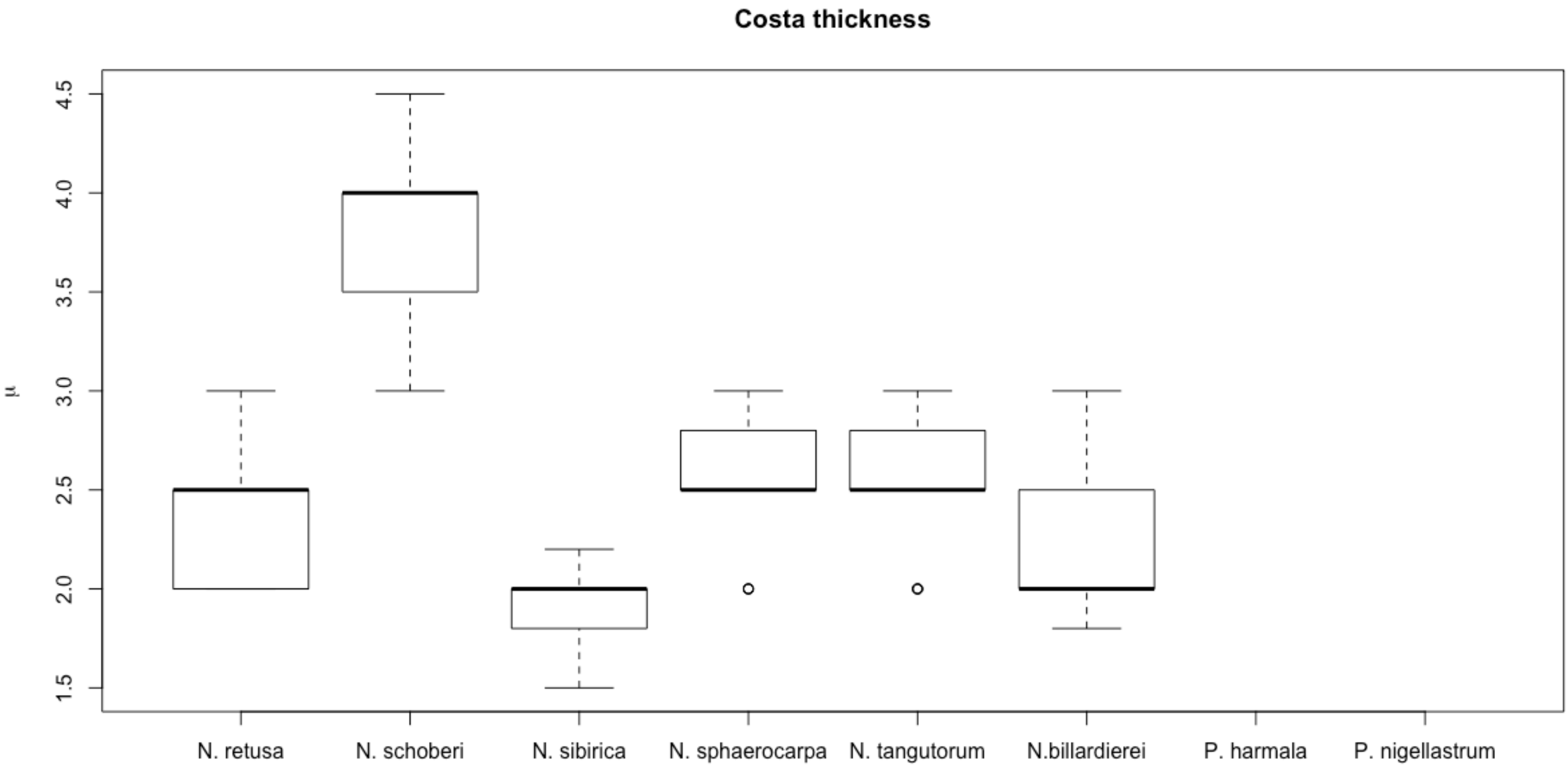
Costa thickness
μ
4.5
4.0
3.5
3.0
2.5
2.0
1.5
N. retusa
N. schoberi
N. sibirica
N. sphaerocarpa
N. tangutorum
N.billardierei
P. harmala
P. nigellastrum

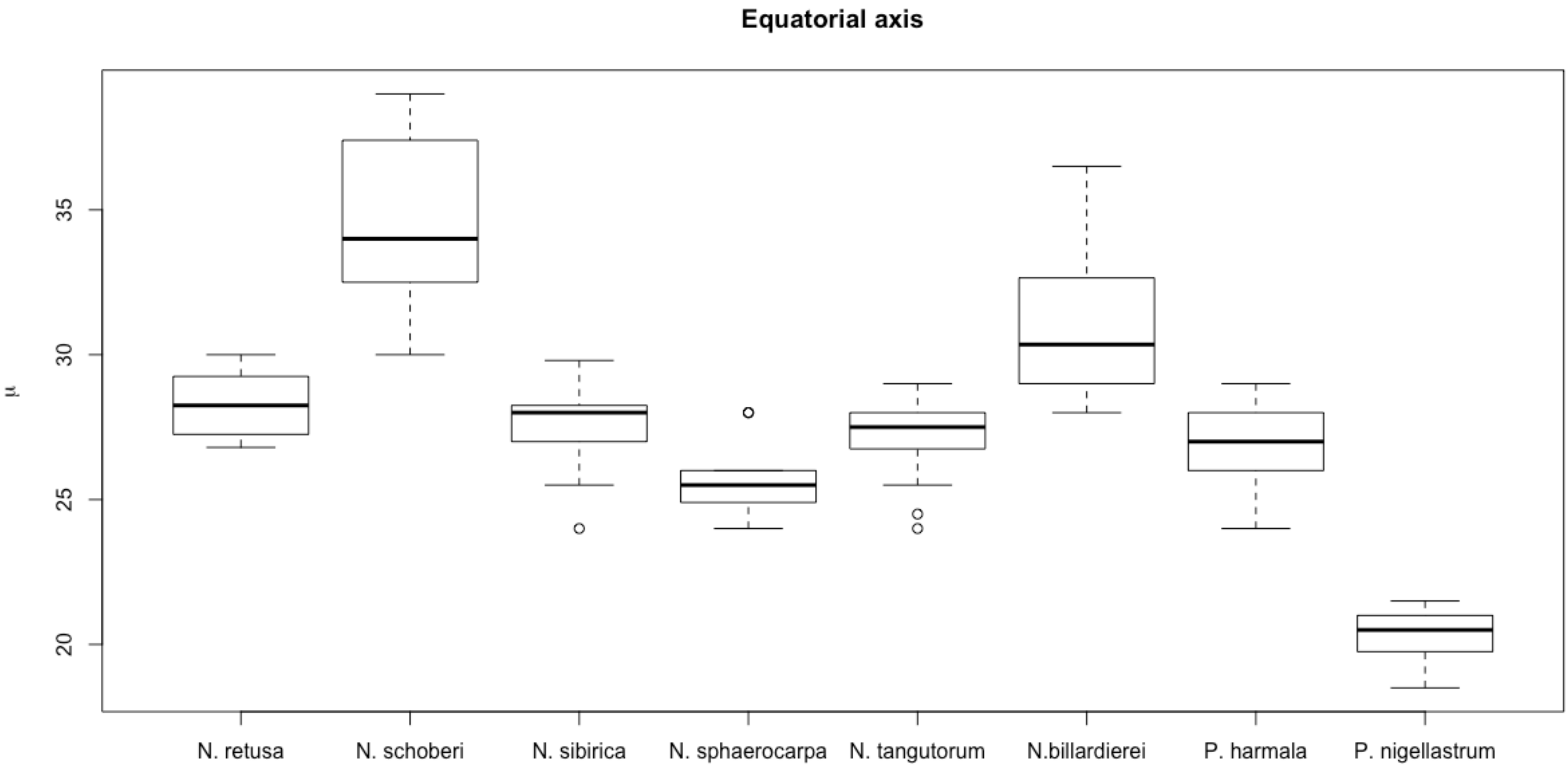

Equatorial axis
μ
20
25
30
35
N. retusa
N. schoberi
N. sibirica
N. sphaerocarpa
N. tangutorum
N.billardierei
P. harmala
P. nigellastrum

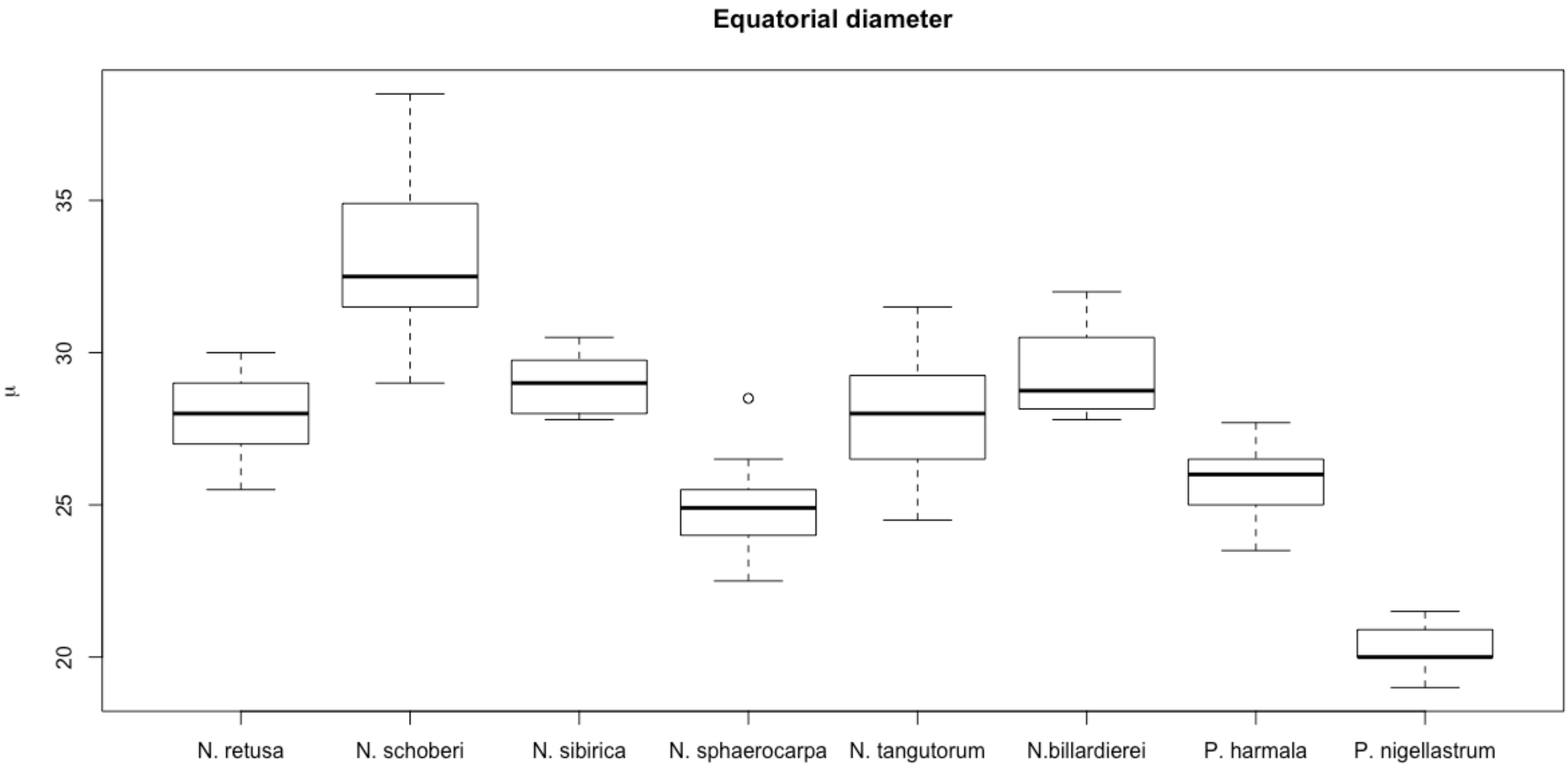
Equatorial diameter
μ
35
30
25
20
N. retusa
N. schoberi
N. sibirica
N. sphaerocarpa
N. tangutorum
N.billardierei
P. harmala
P. nigellastrum

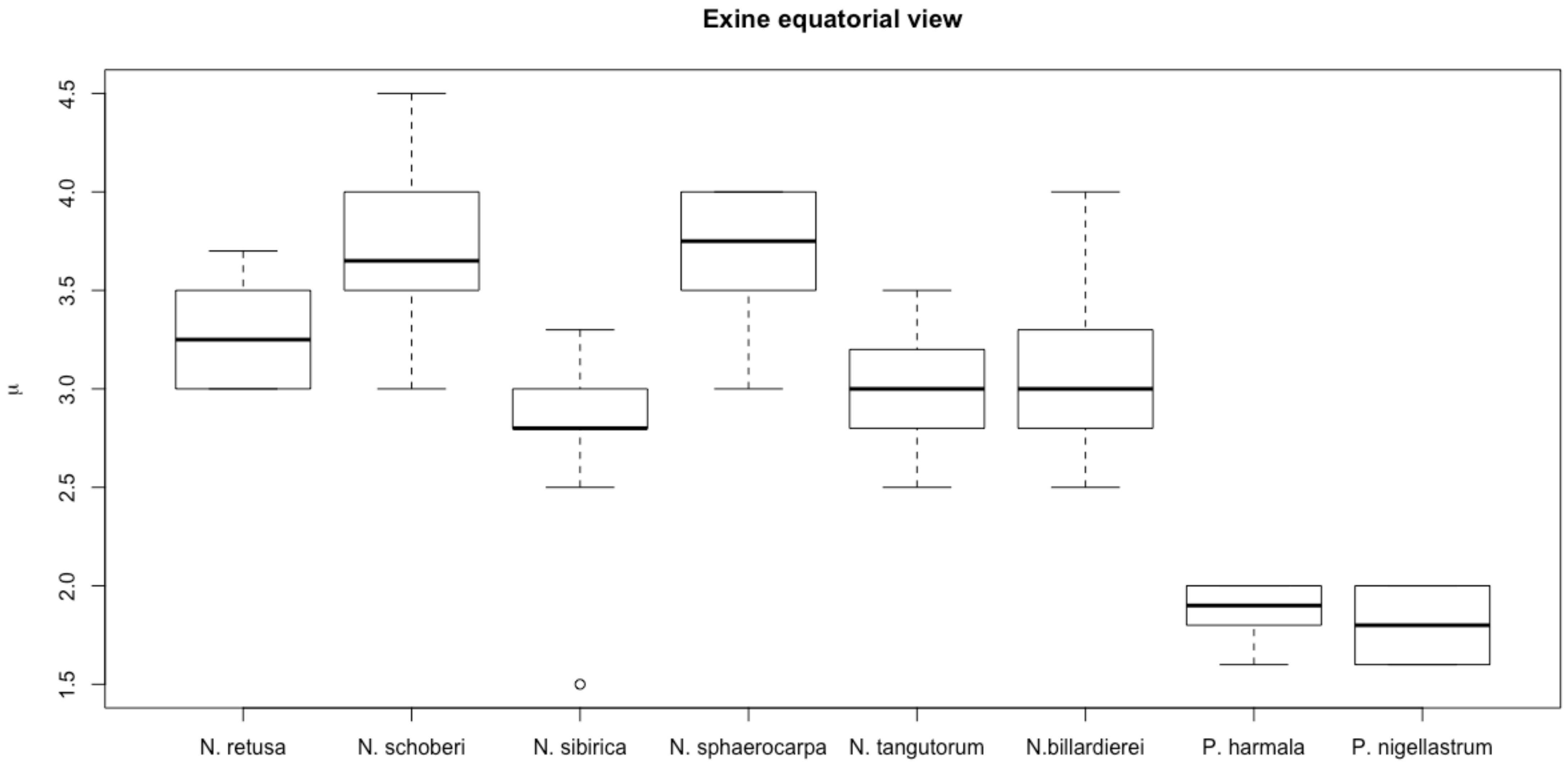

Exine equatorial view
μ
4.5
4.0
3.5
3.0
2.5
2.0
1.5
N. retusa
N. schoberi
N. sibirica
N. sphaerocarpa
N. tangutorum
N.billardierei
P. harmala
P. nigellastrum

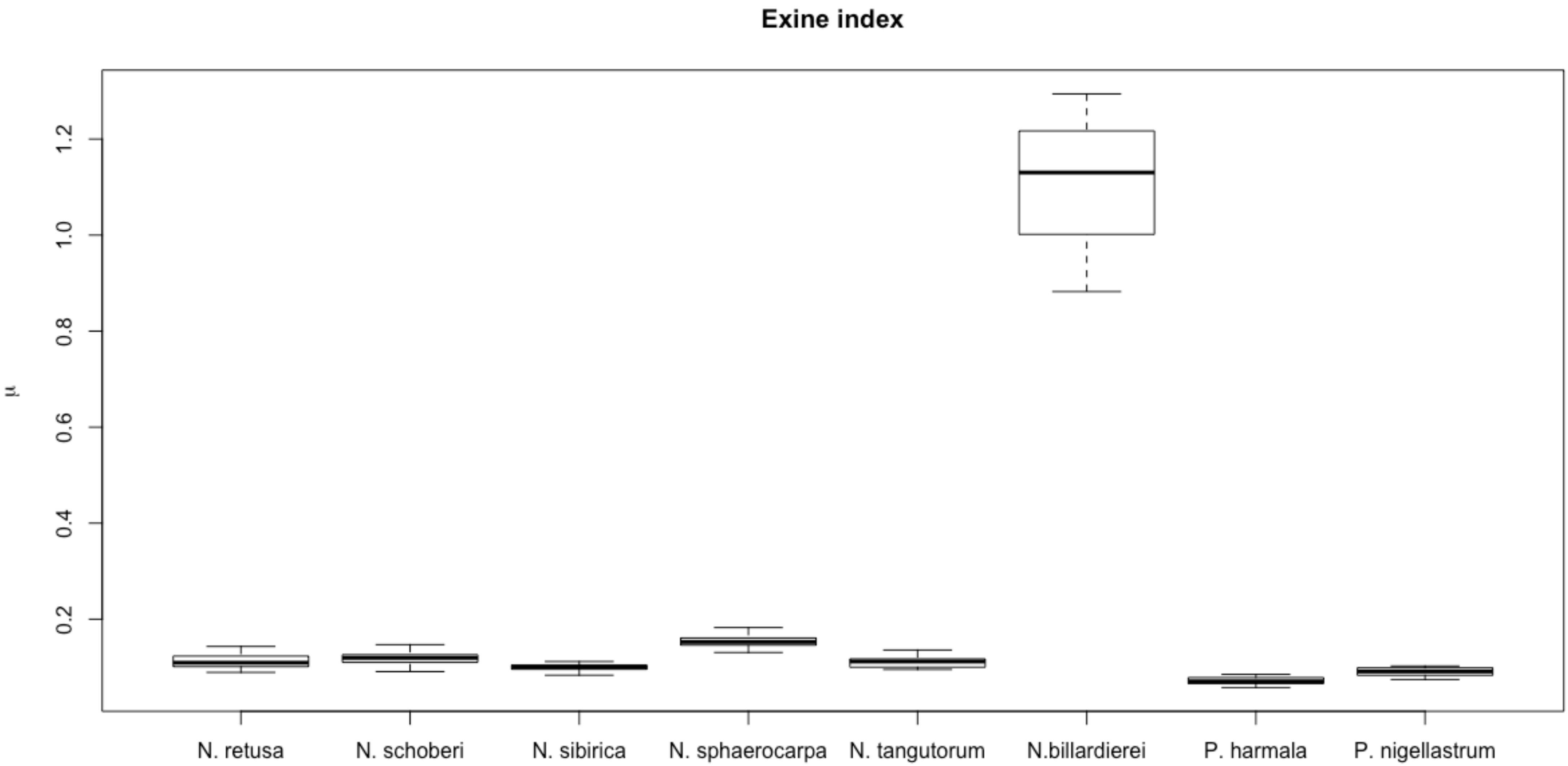

Exine index
µ
0.2
0.4
0.6
0.8
1.0
1.2
N. retusa
N. schoberi
N. sibirica
N. sphaerocarpa
N. tangutorum
N.billardierei
P. harmala
P. nigellastrum

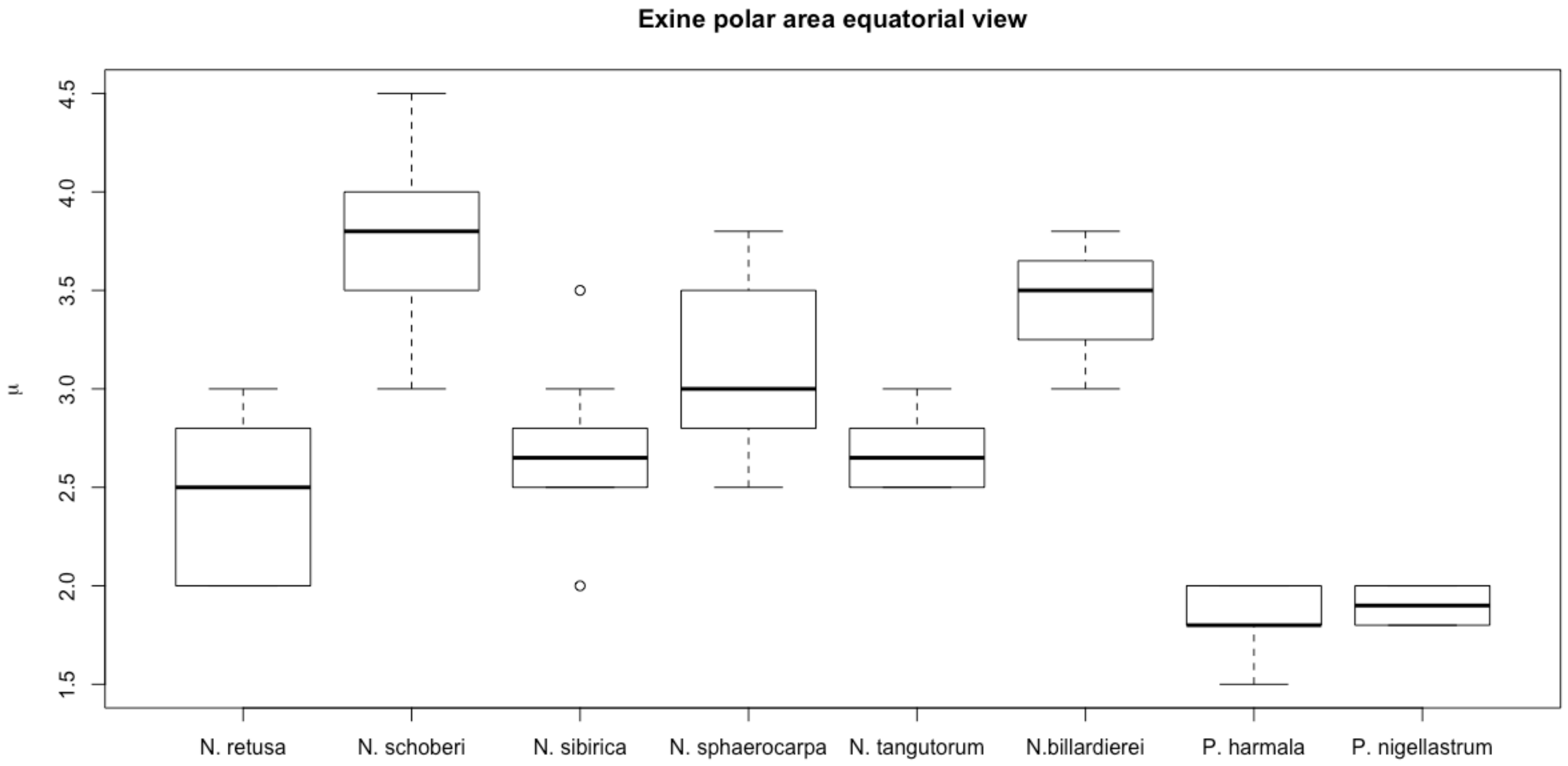
Exine polar area equatorial view
μ
1.5
2.0
2.5
3.0
3.5
4.0
4.5
N. retusa
N. schoberi
N. sibirica
N. sphaerocarpa
N. tangutorum
N.billardierei
P. harmala
P. nigellastrum

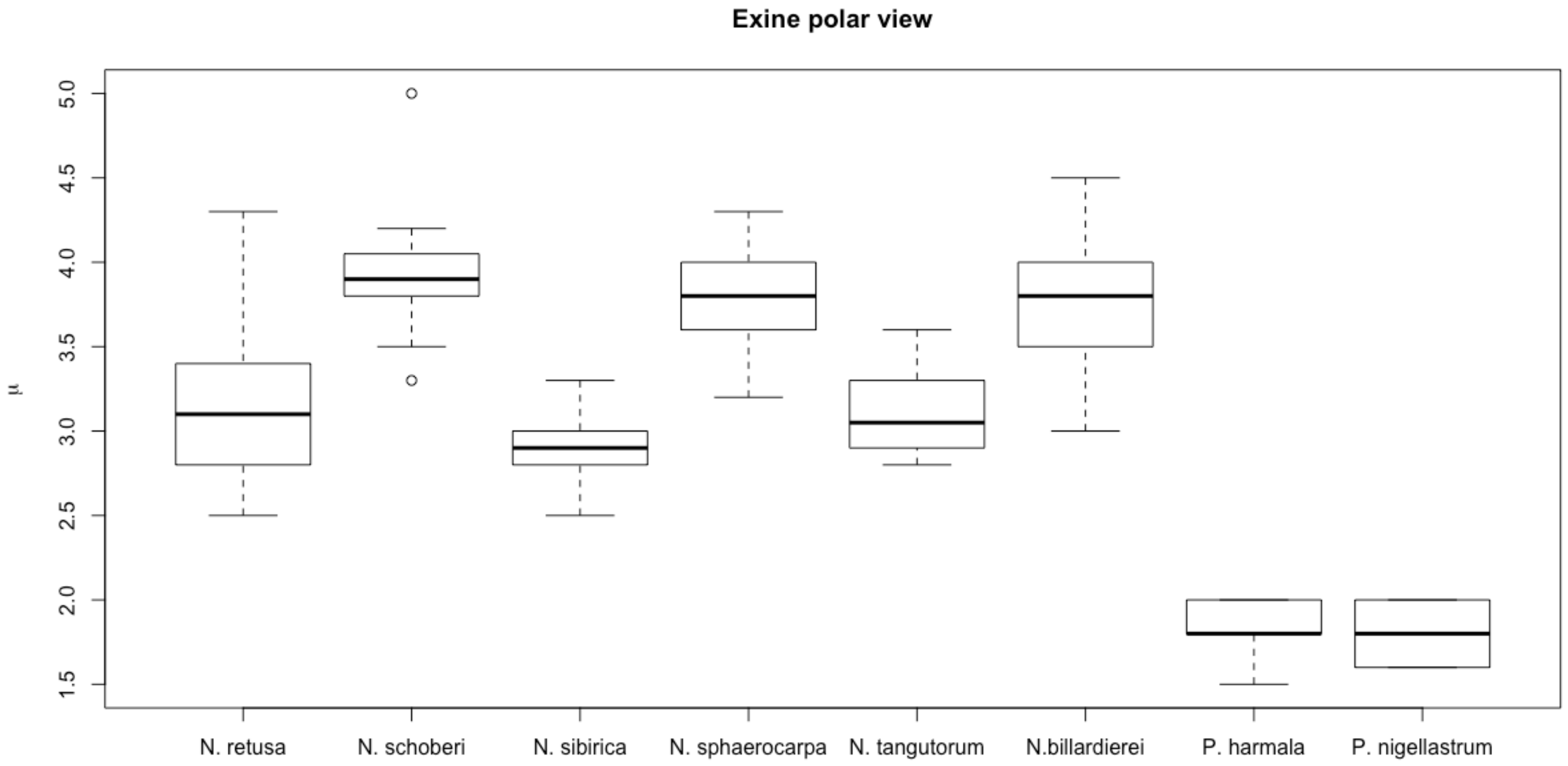

Exine polar view
μ
5.0
4.5
4.0
3.5
3.0
2.5
2.0
1.5
N. retusa
N. schoberi
N. sibirica
N. sphaerocarpa
N. tangutorum
N.billardierei
P. harmala
P. nigellastrum

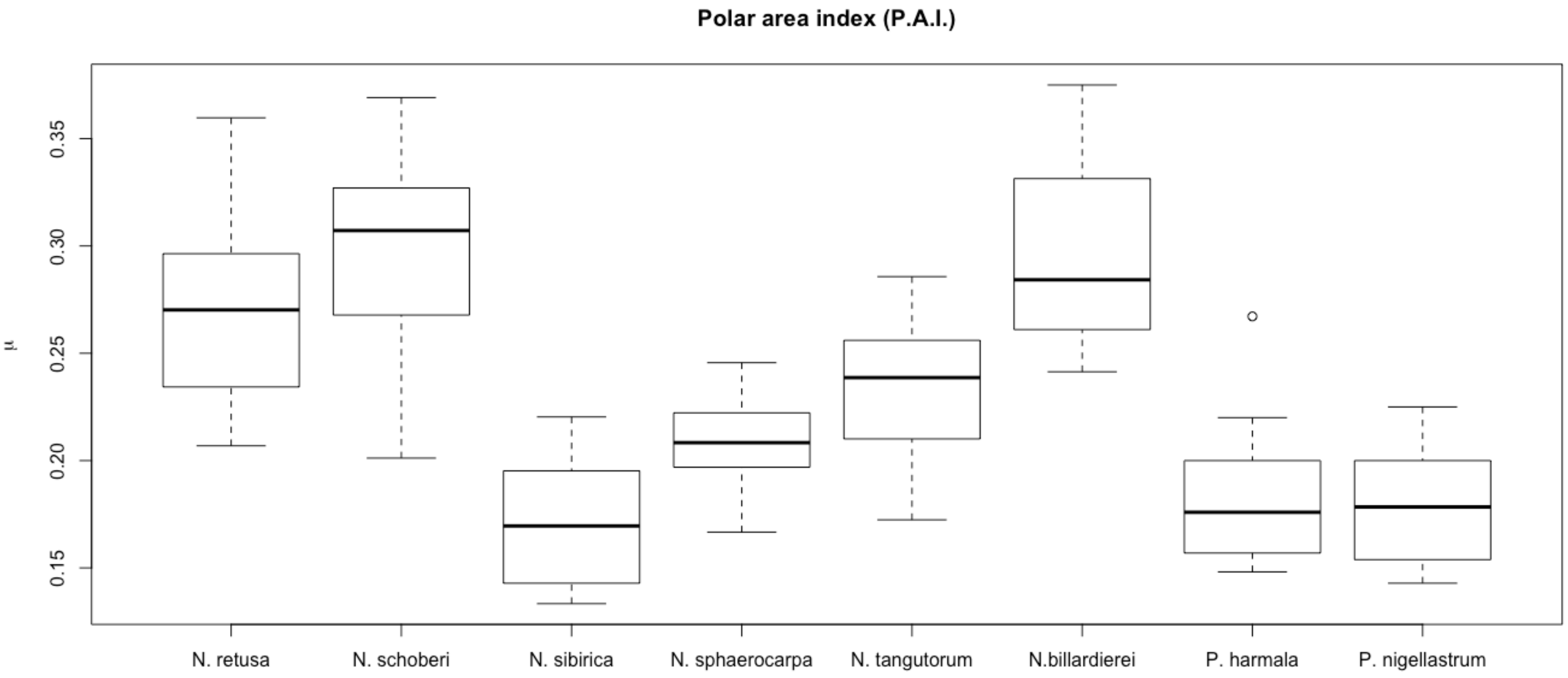

Polar area index (P.A.I.)
μ
0.15
0.20
0.25
0.30
0.35
N. retusa
N. schoberi
N. sibirica
N. sphaerocarpa
N. tangutorum
N.billardierei
P. harmala
P. nigellastrum

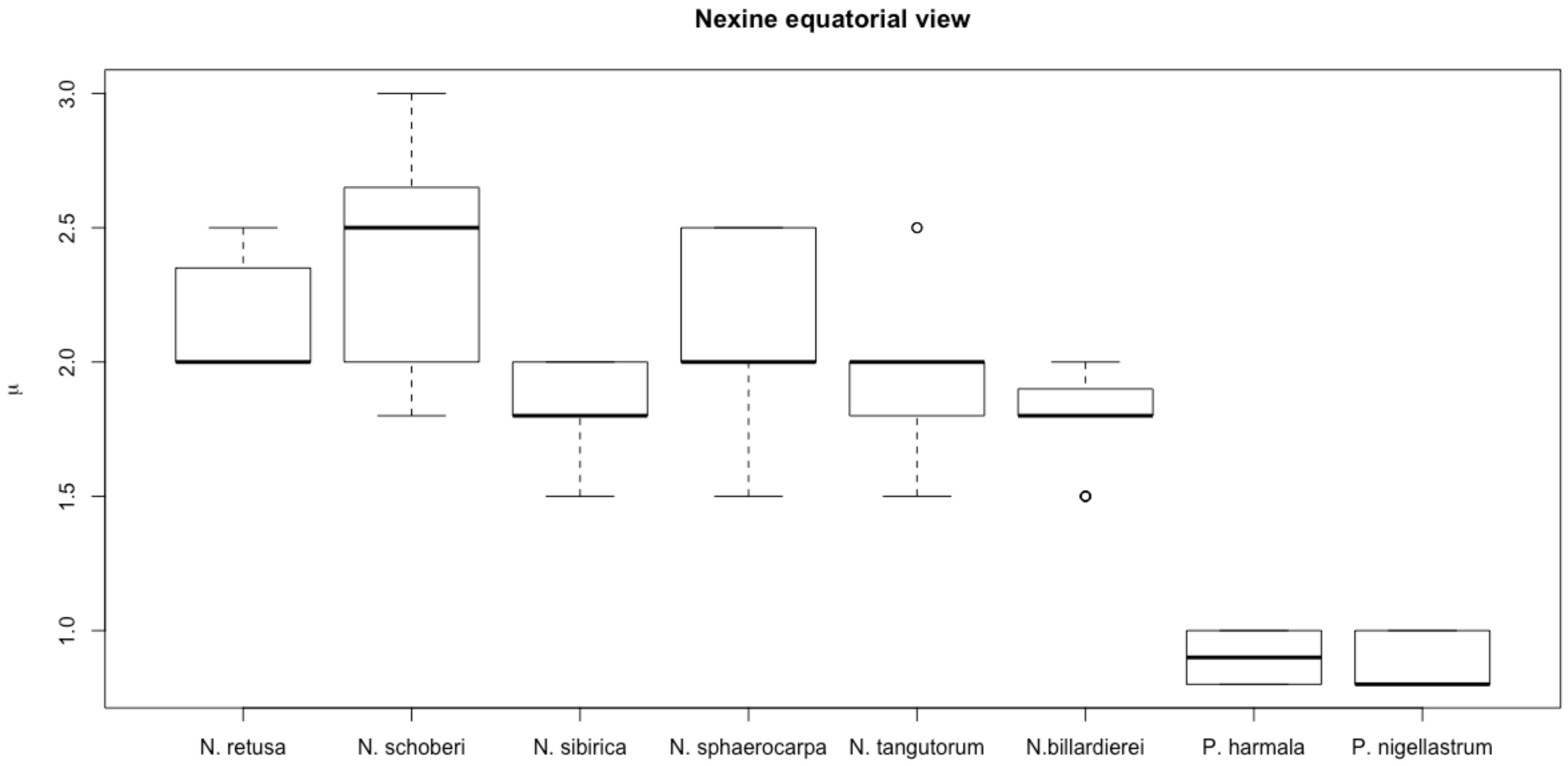
Nexine equatorial view
μ
3.0
2.5
2.0
1.5
1.0
N. retusa
N. schoberi
N. sibirica
N. sphaerocarpa
N. tangutorum
N.billardierei
P. harmala
P. nigellastrum

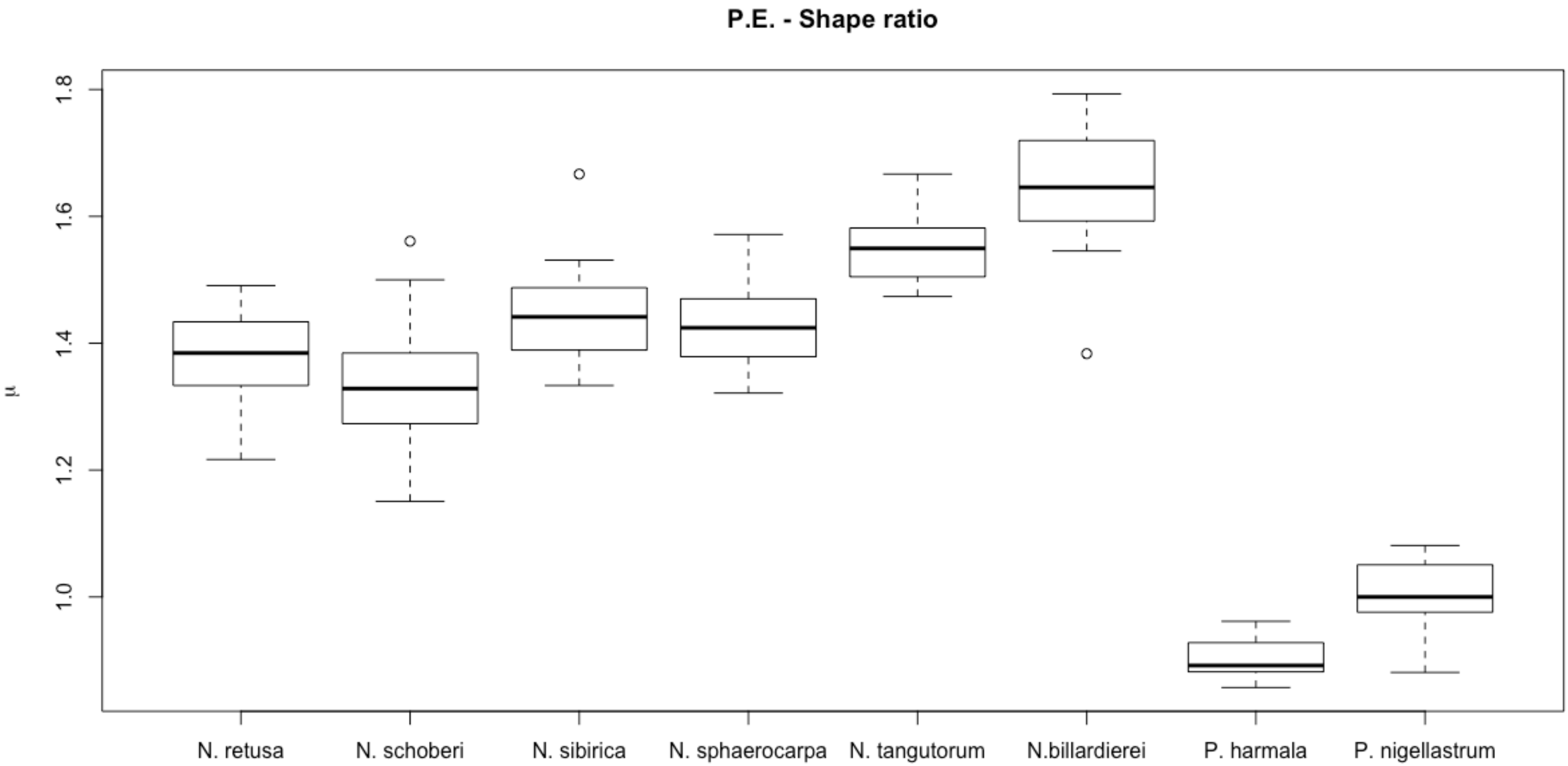

P.E. - Shape ratio
μ
1.0
1.2
1.4
1.6
1.8
N. retusa
N. schoberi
N. sibirica
N. sphaerocarpa
N. tangutorum
N.billardierei
P. harmala
P. nigellastrum

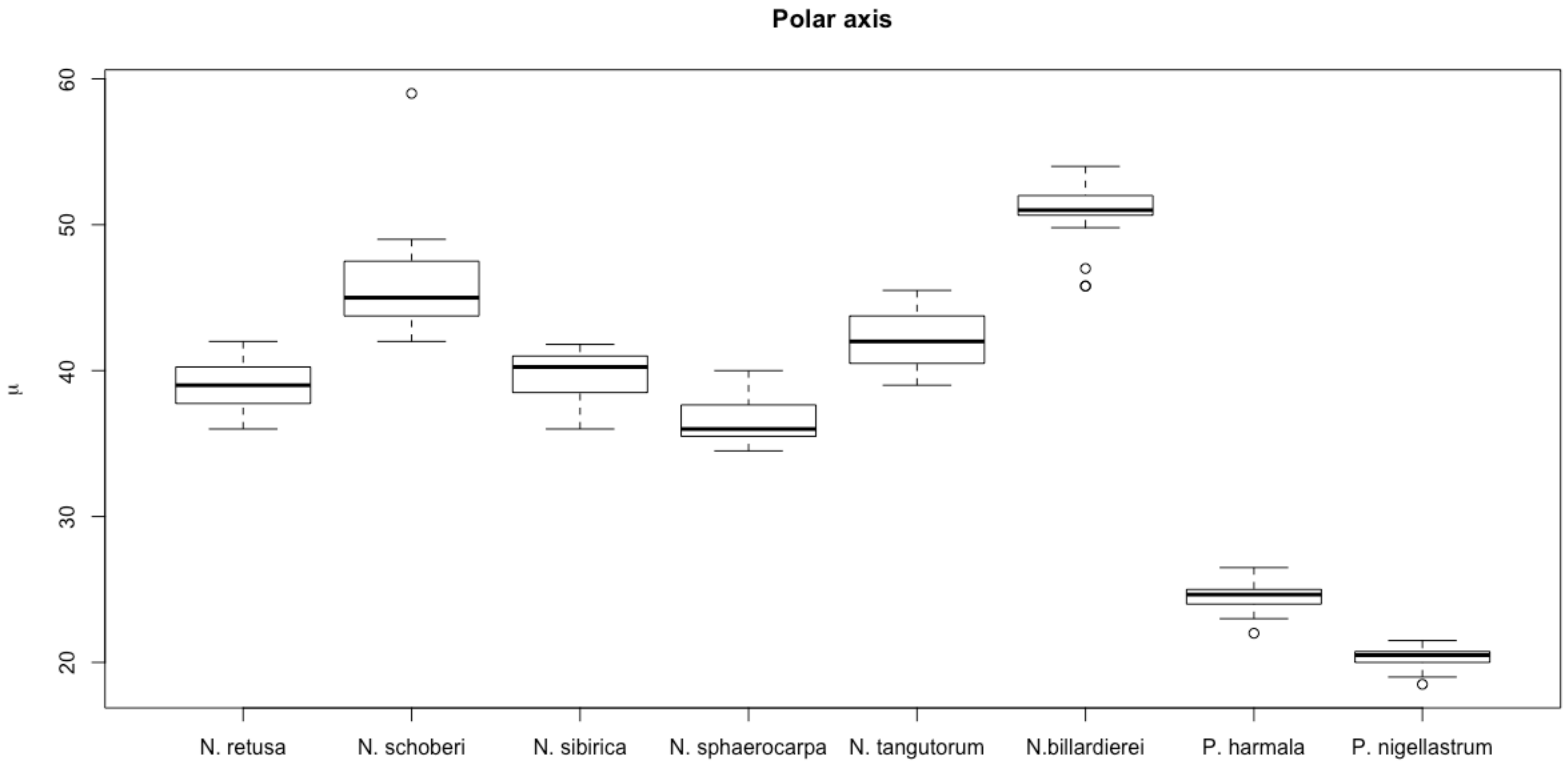
Polar axis
μ
60
50
40
30
20
N. retusa
N. schoberi
N. sibirica
N. sphaerocarpa
N. tangutorum
N.billardierei
P. harmala
P. nigellastrum

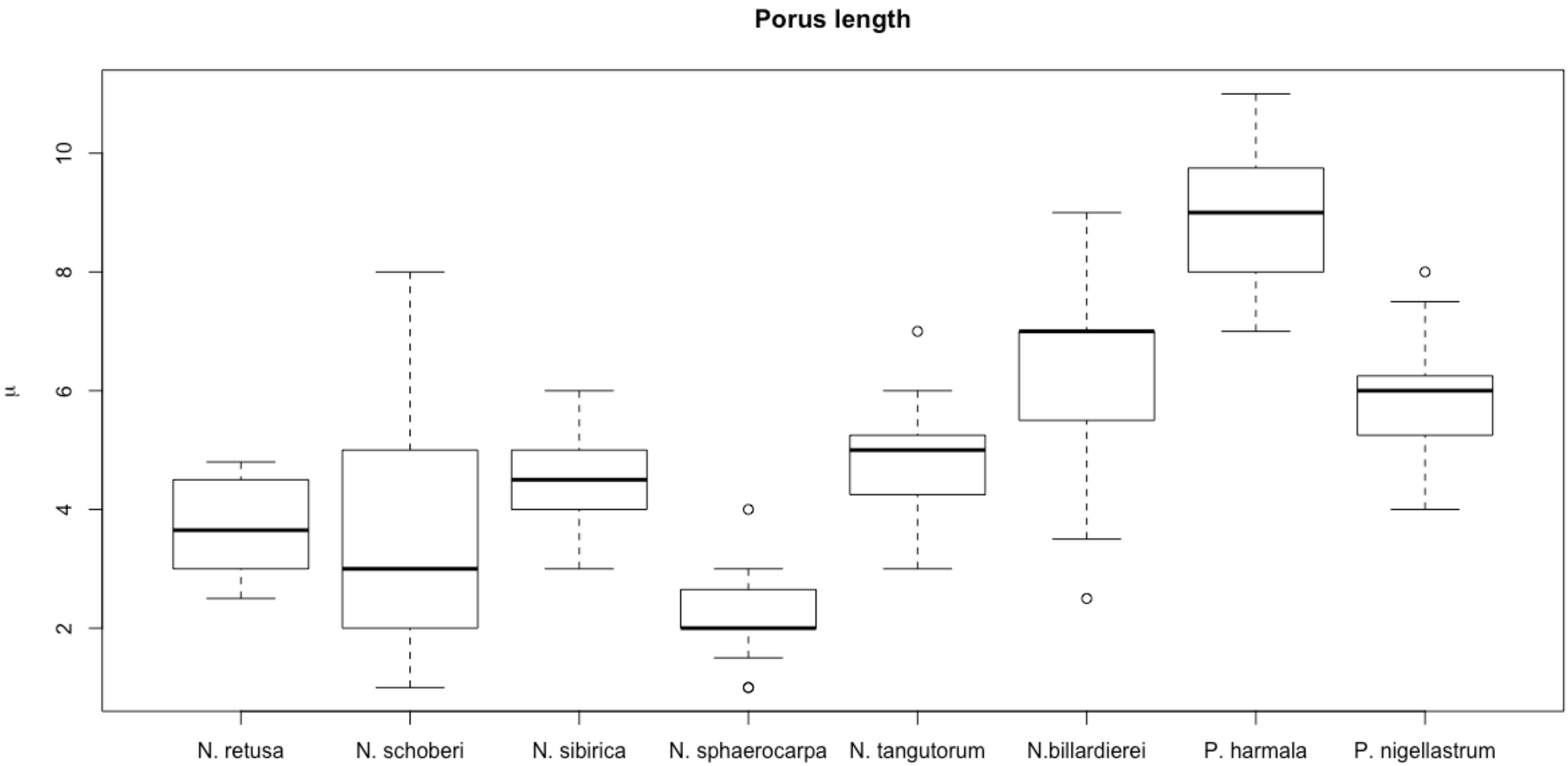

Porus length
μ
2
4
6
8
10
N. retusa
N. schoberi
N. sibirica
N. sphaerocarpa
N. tangutorum
N.billardierei
P. harmala
P. nigellastrum

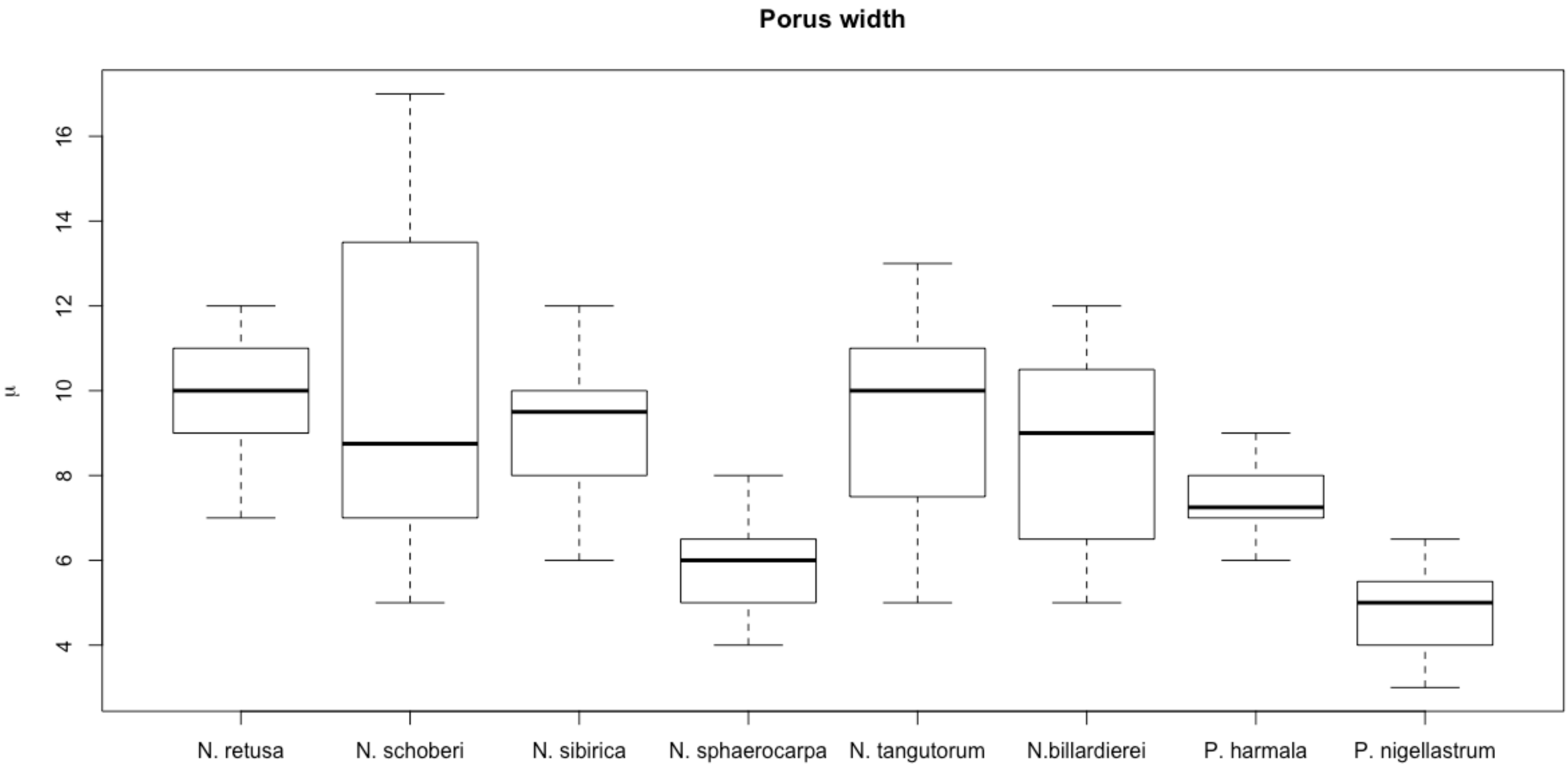

Porus width
μ
4
6
8
10
12
14
16
N. retusa
N. schoberi
N. sibirica
N. sphaerocarpa
N. tangutorum
N.billardierei
P. harmala
P. nigellastrum

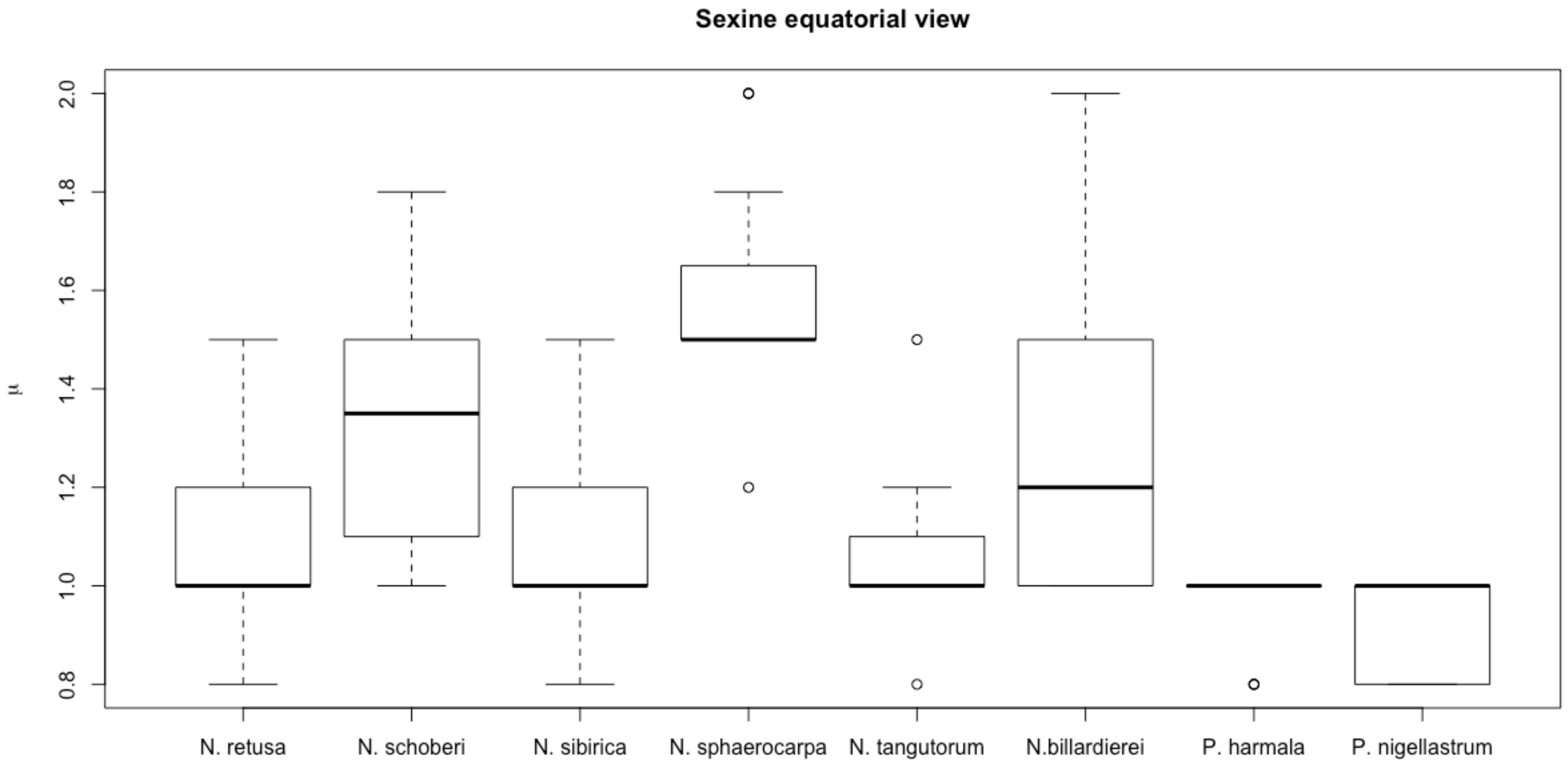

Sexine equatorial view
µ
2.0
1.8
1.6
1.4
1.2
1.0
0.8
N. retusa
N. schoberi
N. sibirica
N. sphaerocarpa
N. tangutorum
N.billardierei
P. harmala
P. nigellastrum
