# Supplementary material for: A novel approach to study the morphology and chemistry of pollen in a phylogenetic context, applied to the halophytic taxon Nitraria L.(Nitrariaceae)
Source: PeerJ. 2018 Jul 19;6:e5055. doi: 10.7717/peerj.5055 (PMC6054868; doi:10.7717/peerj.5055)
Supplement: Supplemental Information 3 [file peerj-06-5055-s007.docx]

**R code from Woutersen et al.** **Pollen morphology and pollen chemistry as identifiers of Nitrariaceae and implications for historical records of the steppe–desert taxon *Nitraria***

#install packages

library(FD)

library(ape)

library(phytools)

library(vegan)

library(baseline)

library(prospectr)

library(class)

#Load data

nit.tree <- read.nexus("nitrariaceae7Zhang.tre") #Maximum credibility tree from Zhang et al 2015

nit.morph <- read.table("Morphology_characterCodings.txt", header = T, row.names = 1) #Morphology

nit.chem <- read.table("FTIR_spectralData.txt", header = T, row.names = 1) #Chemistry

#Create vectors of taxon names for each specimen (used in plotting later)

taxa.chem <- as.factor(unlist(strsplit(rownames(nit.chem), "[.]"))[seq(1, nrow(nit.chem)*2, by = 2)])

taxa.morph <- as.factor(unlist(strsplit(rownames(nit.morph), "[.]"))[seq(1, nrow(nit.morph)*2, by = 2)])

##Phylogeny

nit.tree$tip.label[1] <- "Nitraria_billardierei" #update spelling

plot(nit.tree, label.offset = 1.5)

axisPhylo()

#Prune tree to taxa in morphology dataset

nit.tree$tip.label[3] <- "Nitraria_retusa"

nit.tree.morph <- drop.tip(nit.tree, setdiff(nit.tree$tip.label, taxa.morph))

plot(nit.tree.morph, label.offset = 1.5)

axisPhylo()

##Morphology

#Compute gower distance

nit.gow <- gowdis(nit.morph)

#NMDS

nit.nmds <- metaMDS(nit.gow)

nit.nmds$stress

#[1] 0.1184658

#Plot up

nit.morph.cols <- c("red", "orangered", "orange", "maroon", "orchid", "pink", "darkblue", "blue")

nit.morph.points <- c(0:5, 17, 19)

taxon.names.morph <- c("N. billardierei", "N. retusa", "N. schoberi",

"N. sibirica", "N. sphaerocarpa", "N. tangutorum",

"P. harmala", "P. nigellastrum")

nit.nmds.scores <- nit.nmds$points

plot(nit.nmds.scores[,1],

nit.nmds.scores[,2],

ylim = c(-0.15, 0.2),

type = "p", xlab = "NMDS 1", ylab = "NMDS 2",

las=1,

pch = nit.morph.points[as.numeric(taxa.morph)],

col = nit.morph.cols[as.numeric(taxa.morph)],

main = "")

legend("topright", legend = taxon.names.morph,

pch = nit.morph.points[as.numeric(unique(taxa.morph))],

col = nit.morph.cols[as.numeric(unique(taxa.morph))],

text.font = 3, y.intersp = 0.8)

#Phylomorphospace from mean NMDS axis scores

nit.nmds.scores.means <- aggregate(nit.nmds.scores, by = list(taxa.morph),

FUN = mean)[,-1]

rownames(nit.nmds.scores.means) <- nit.tree.morph$tip.label

phylomorphospace(nit.tree.morph, nit.nmds.scores.means[,c(1,2)],

ylim = c(-0.15, 0.2),

xlab = "NMDS 1", ylab = "NMDS 2",

label = "horizontal")

##Chemistry

wavenumbers <- as.numeric(unlist(strsplit(colnames(nit.chem), "X"))[seq(2, 3450, by = 2)])

#Baseline correct with 2nd order polynomial

nit.chem.base <- baseline(as.matrix(nit.chem),

method='modpolyfit', deg=2)

nit.chem.cor <- getCorrected(nit.chem.base)

nit.chem.cor <- as.data.frame(nit.chem.cor,

row.names = rownames(nit.chem))

colnames(nit.chem.cor) <- wavenumbers

#Z-score standardisation

nit.chem.cor.stand <- t(scale(t(nit.chem.cor)))

#Check

apply(nit.chem.cor.stand, 1, mean)

apply(nit.chem.cor.stand, 1, var)

#Plot up mean spectrum for each species

nit.chem.taxa <- aggregate(nit.chem.cor.stand, by = list(taxa.chem),

FUN = mean)[,-1]

rownames(nit.chem.taxa) <- unique(taxa.chem)

taxon.names.chem <- c("N. billardierei", "N. retusa", "N. schoberi",

"N. sibirica", "N. sphaerocarpa", "N. tangutorum",

"P. nigellastrum")

peaks <- c(3390, 2925, 2850, 1710, 1600, 1510, 1440, 1370, 1170, 1030)

par(mar = c(3.5,2,2,1)+0.1)

plot(wavenumbers, nit.chem.taxa[1,],

xlim = c(4000, 700), ylim = c(-1, 32),

type = "n", las = 1,

yaxt = "n", xaxt = "n",

xlab = "", ylab = "")

for(i in 1:length(peaks)) {

abline(v = peaks[i], col = "grey50", lty = 3)

}

lines(wavenumbers, nit.chem.taxa[1,]+30)

lines(wavenumbers, nit.chem.taxa[2,]+25)

lines(wavenumbers, nit.chem.taxa[3,]+20)

lines(wavenumbers, nit.chem.taxa[4,]+15)

lines(wavenumbers, nit.chem.taxa[5,]+10)

lines(wavenumbers, nit.chem.taxa[6,]+5)

lines(wavenumbers, nit.chem.taxa[7,])

axis(1, lwd = 0, lwd.ticks = 1, tcl = -0.5,

mgp = c(3, 0.8, 0),

las = 1)

title(ylab = "Absorbance", line = 1)

title(xlab = "Wavenumber (cm-1)", line = 2.5)

#Add on taxon names

for(i in length(taxon.names.chem):1) {

text(x = 4130, y = 5*(9-i)-8.5, labels = taxon.names.chem[i],

pos = 4, font = 3)

}

#Add on peak positions

for(i in 1:length(peaks)) {

mtext(text = peaks[i], side = 3, at = peaks[i], cex = 0.7)

}

#Classification

#No smoothing or derivatives

m0 <- knn.cv(train = nit.chem.cor.stand,

cl = taxa.chem,

k = 1)

(m0.success <- sum(m0 == taxa.chem)/length(taxa.chem))

#[1] 0.8392857

#Smoothing, no derivatives

m0.sg.success <- numeric(20)

for (i in 1:length(m0.sg.success)){

nit.chem.sg <- savitzkyGolay(nit.chem.cor.stand, p = 3, w = 2*i+3, m = 0)

m0.sg <- knn.cv(train = nit.chem.sg,

cl = taxa.chem,

k = 1)

m0.sg.success[i] <- sum(m0.sg == taxa.chem)/length(taxa.chem)

}

max(m0.sg.success)

#[1] 0.8482143

2*which(m0.sg.success == max(m0.sg.success))+3

#[1] 21 23 25 27 29 31 33 35 37 39 41 43

#Smoothing, 1st derivative

m1.sg.success <- numeric(20)

for (i in 1:length(m1.sg.success)){

nit.chem.m1.sg <- savitzkyGolay(nit.chem.cor.stand, p = 3, w = 2*i+3, m = 1)

m1.sg <- knn.cv(train = nit.chem.m1.sg,

cl = taxa.chem,

k = 1)

m1.sg.success[i] <- sum(m1.sg == taxa.chem)/length(taxa.chem)

}

max(m1.sg.success)

#[1] 0.9553571

2*which(m1.sg.success == max(m1.sg.success))+3

#[1] 9 11 13 15 17 19 21 23 25 27

#Smoothing, 2nd derivative

m2.sg.success <- numeric(20)

for (i in 1:length(m2.sg.success)){

nit.chem.m2.sg <- savitzkyGolay(nit.chem.cor.stand, p = 3, w = 2*i+3, m = 2)

m2.sg <- knn.cv(train = nit.chem.m2.sg,

cl = taxa.chem,

k = 1)

m2.sg.success[i] <- sum(m2.sg == taxa.chem)/length(taxa.chem)

}

max(m2.sg.success)

#[1] 0.9642857

2*which(m2.sg.success == max(m2.sg.success))+3

#[1] 37 39

#Proceed with 2nd derivative and w = 37

nit.chem.m2.sg <- savitzkyGolay(nit.chem.cor.stand, p = 3, w = 37, m = 2)

#PCA on individual spectra

nit.chem.m2.sg.pca <- prcomp(nit.chem.m2.sg)

#Importance of PCA axes

summary(nit.chem.m2.sg.pca)

par(mar = c(5,4,2,2) + 0.1)

barplot(summary(nit.chem.m2.sg.pca)[[6]][3,1:10],

ylim = c(0, 1), las = 1, xlab = "PC axes (10 of 112)",

ylab = "Cumulative proportion of variance",

main = "")

abline(h = 0.9, lty = 2) #90% of variance explained with 6 axes

#First two explain 67%, first three explain 80%

#Plot up

taxon.names.chem <- c("N. billardierei", "N. retusa", "N. schoberi",

"N. sibirica", "N. sphaerocarpa", "N. tangutorum",

"P. nigellastrum")

nit.chem.cols <- c("red", "orangered", "orange", "maroon", "orchid", "pink", "blue")

nit.chem.points <- c(0:5, 19)

nit.chem.m2.sg.pca.scores <- as.data.frame(nit.chem.m2.sg.pca$x)

plot(nit.chem.m2.sg.pca.scores[,1],

nit.chem.m2.sg.pca.scores[,2],

xlim = c(-0.04, 0.02), ylim = c(-0.02, 0.02),

type = "p", xlab = "PCA 1 (47%)", ylab = "PCA 2 (20%)",

las=1, pch = nit.chem.points[as.numeric(taxa.chem)],

col = nit.chem.cols[as.numeric(taxa.chem)],

main = "")

legend("bottomleft", legend = taxon.names.chem,

pch = nit.chem.points[as.numeric(unique(taxa.chem))],

col = nit.chem.cols[as.numeric(unique(taxa.chem))],

text.font = 3, y.intersp = 0.8)

#PCA/phylochemospace on taxon mean spectra

#Calculate mean spectra

nit.chem.m2.sg.means <- aggregate(nit.chem.m2.sg, by = list(taxa.chem),

FUN = mean)[,-1]

rownames(nit.chem.m2.sg.means) <- unique(taxa.chem)

#Prune tree to taxa chem dataset

nit.tree.chem <- drop.tip(nit.tree, setdiff(nit.tree$tip.label, rownames(nit.chem.m2.sg.means)))

#PCA

nit.chem.m2.sg.means.pca <- prcomp(nit.chem.m2.sg.means)

#Importance of PCA axes

summary(nit.chem.m2.sg.means.pca)

barplot(summary(nit.chem.m2.sg.means.pca)[[6]][3,],

ylim = c(0, 1), las = 1, xlab = "PC axes",

ylab = "Cumulative proportion of variance",

main = "")

abline(h = 0.9, lty = 2) #>90% of variance explained with 3 axes

#First two explain 83%

#Extract scores and plot up

nit.chem.m2.sg.means.pca.scores <- as.data.frame(nit.chem.m2.sg.means.pca$x)

phylomorphospace(nit.tree.chem, nit.chem.m2.sg.means.pca.scores[,c(1,2)],

xlim = c(-0.03, 0.02), ylim = c(-0.015, 0.01),

xlab="PCA 1, 56% variance",

ylab="PCA 2, 27% variance",

label = "horizontal")

phylomorphospace(nit.tree.chem, nit.chem.m2.sg.means.pca.scores[,c(1,3)],

xlim = c(-0.03, 0.02), ylim = c(-0.01, 0.01),

xlab="PCA 1, 56% variance",

ylab="PCA 3, 11% variance",

label = "horizontal")
